# SMURF1 attenuates endoplasmic reticulum stress by promoting the degradation of KEAP1 to activate NRF2 antioxidant pathway

**DOI:** 10.1038/s41419-023-05873-2

**Published:** 2023-06-14

**Authors:** Lei Dong, Mengchuan Xu, Yang Li, Wanting Xu, Chengwei Wu, Hanfei Zheng, Zhenyu Xiao, Guochen Sun, Lei Ding, Xiaobo Li, Wenming Li, Liying Zhou, Qin Xia

**Affiliations:** 1 Key Laboratory of Molecular Medicine and Biological Diagnosis and Treatment (Ministry of Industry and Information Technology), School of Life Science, Beijing Institute of Technology, Beijing, 100081 China; 2 Department of Neurosurgery, The First Medical Centre, Chinese PLA General Hospital, Beijing, 100853 China; 3 Key Laboratory of Carcinogenesis and Translational Research (Ministry of Education/Beijing), Department of Anesthesiology, Peking University Cancer Hospital & Institute, Beijing, 100142 China; 4 BeiJing Tide Pharmaceutical Co. LTD, BeiJing, 102600 China

**Keywords:** Cell biology, Diseases

## Abstract

Cancer cells consistently utilize the unfolded protein response (UPR) to encounter the abnormal endoplasmic reticulum (ER) stress induced by the accumulation of misfolded proteins. Extreme activation of the UPR could also provoke maladaptive cell death. Previous reports have shown that NRF2 antioxidant signaling is activated by UPR and serves as noncanonical pathway to defense and reduce excessive ROS levels during ER stress. However, the mechanisms of regulating NRF2 signaling upon ER stress in glioblastoma have not been fully elucidated. Here we identify that SMURF1 protects against ER stress and facilitates glioblastoma cell survival by rewiring KEAP1-NRF2 pathway. We show that ER stress induces SMURF1 degradation. Knockdown of SMURF1 upregulates IRE1 and PERK signaling in the UPR pathway and prevents ER-associated protein degradation (ERAD) activity, leading to cell apoptosis. Importantly, SMURF1 overexpression activates NRF2 signaling to reduce ROS levels and alleviate UPR-mediated cell death. Mechanistically, SMURF1 interacts with and ubiquitinates KEAP1 for its degradation (NRF2 negative regulator), resulting in NRF2 nuclear import. Moreover, SMURF1 loss reduces glioblastoma cell proliferation and growth in subcutaneously implanted nude mice xenografts. Taken together, SMURF1 rewires KEAP1-NRF2 pathway to confer resistance to ER stress inducers and protect glioblastoma cell survival. ER stress and SMURF1 modulation may provide promising therapeutic targets for the treatment of glioblastoma.

## Introduction

Glioblastoma is the most malignant primary brain tumor, characterized by high heterogeneity, resistance and relapse [1, 2]. The current treatment strategies including maximal-safe surgical resection and adjuvant radiation therapy with alkylating agent temozolomide (TMZ) treatment have shown limited survival benefits, with a median survival rate about 14.6 months [3, 4]. Glioblastoma cells have a high metabolic rate and produce high levels of reactive oxide species (ROS), which further disturbs the protein folding capacity [5]. Endoplasmic reticulum (ER) stress due to the accumulation of unfolded or misfolded proteins is present highly in glioblastoma [6, 7]. The mechanisms are involved in response to ER stress by the three UPR sensors, including activating transcription factor 6 alpha (ATF6α), inositol requiring enzyme 1 (IRE1) and protein kinase R (PKR)-like ER kinase (PERK). ER chaperone BiP/GRP78 constitutively binds to the three UPR sensors, resulting in their inactivation at the basal level. Under ER stress, BiP/GRP78 dissociates from the UPR sensors to activate IRE1, PERK and ATF6α by oligomerization and trans-autophosphorylation or secretion of ATF6α [8, 9]. UPR response is an adaptive mechanism to restore ER homeostasis through multiple pathways, including attenuating transcriptional signal, alleviating the accumulation of misfolded proteins via ER-associated protein degradation (ERAD) system and recycling of misfolded proteins through the induction of autophagy [8, 10–12]. Accumulating evidences have indicated that a high level of basal UPR is frequently found in primary human tumors including glioblastoma, and adaptive UPR promotes cancer survival upon adverse environments [13–15]. However, the aberrant activation of UPR also triggers cell death under the unresolved and extreme ER stress conditions [16, 17]. For instance, arginosuccinate synthase 1 treatment suppresses tumor progression and triggers pro-apoptotic ER stress responses in hepatocellular carcinoma through the hyperactivation of PERK-eukaryotic translation initiation factor 2α (eIF2α)-activating transcription factor 4 (ATF4)-C/EBP homologous protein (CHOP) arm of the UPR [18].

Misfolded protein accumulation and aggregation induce excessive production of ROS, which can activate nuclear factor erythroid 2-related factor 2 (NRF2). Many lines of evidences have shown that the activation of NRF2 pathway suppresses ER stress-induced apoptosis by regulating antioxidant synthesis and ROS eliminating enzymes expression [19, 20]. Under normal conditions, NRF2 is sequestered in the cytoplasm and degraded by its negative factor Kelch-like ECH-associated protein 1 (KEAP1) [21]. Whereas upon oxidative stress, the canonical KEAP1-NRF2 pathway activation is mediated by the release of NRF2 from KEAP1, then NRF2 translocates into the nucleus to activate antioxidant genes such as NAD(P)H quinone dehydrogenase 1 (NQO1) and HMOX1/HO-1 heme oxygenase 1 (HO1) [14, 22]. The noncanonical KEAP1-NRF2 pathway activation is mediated by p62/SQSTM1, an autophagy receptor protein that competitively binds with and degrades KEAP1 to activate NRF2 [22–24]. Indeed, NRF2-p62 system and selective autophagy are vital in tolerance of tumor microenvironmental stress [25, 26]. ER stress inducing agents, such as ER Ca^2+^ pump inhibitor TG (Thapsigargin) and the N-glycosylation inhibitor TM (Tunicamycin), increase ROS production and trigger tumor cell apoptosis [20]. NRF2 could be phosphorylated by PERK during ER stress, further triggering NRF2 dissociation from KEAP1 and induction of protective antioxidant response [27]. In addition, PERK-dependent activation of NRF2 attenuates accumulation of ROS triggering oxidative DNA damage and contributes to redox homeostasis and cell survival [28, 29]. Importantly, studies have reported that activation of NRF2 induces several components of the transcriptional UPR target genes, including XBP1 and ATF6α, to maintain ER integrity and protein homeostasis [30]. NRF2 interacts and activates ATF4 to induce the target genes expression to survive proteotoxic stress [31, 32]. Therefore, NRF2-UPR axis serves as a bidirectional signal for maintaining ER homeostasis. Accumulating evidences have suggested that sustained activation of NRF2 also induces pro-survival genes that promote cancer cell proliferation and chemoresistance [33]. Recent studies have shown that aberrantly high expression of NRF2 signaling is found in glioblastoma and promotes tumor cell mesenchymal transition, invasion and tumorigenesis [34]. However, the mechanisms of regulating NRF2 signaling in glioblastoma have not been well defined.

The HECT-type ubiquitin ligase (E3) Smad ubiquitination regulatory factor 1 (SMURF1) belongs to the Nedd4 family and mediates multiple biological processes, including cell growth and migration, and several physiological functions in bone formation, embryonic development, and tumorigenesis [35–39]. Our previous studies have demonstrated that SMURF1 is hyperactivated in glioblastoma and promotes tumor growth by ubiquitination and degradation of tumor suppresser phosphatase and tensin homolog (PTEN) [40]. SMURF1 also modulates the K63-linked polyubiquitination of mutant superoxide dismutase 1 (SOD1) to promote aggresome formation and p62-dependent autophagic degradation [41]. Of importance, SMURF1 is found to facilitate selective autophagy by recruitment of microtubule-associated protein 1 light chain 3 (MAP1LC3/LC3) or ubiquitination of UV radiation resistance associated (UVRAG), an important regulator of mammalian macroautophagy/autophagy [42, 43]. However, the roles and mechanisms of SMURF1 in certain physiological or pathological stress are poorly known. Previous studies have reported that overexpression of SMURF1 partially reverses the effect of ER stress inducer by ubiquitination and proteasomal degradation of ER-localized protein wolfram syndrome protein (WFS1), which downregulates the expression level of ATF6α [44]. Altogether, SMURF1 as a newly recognized mediator of selective autophagy may be involved in the regulation of NRF2. But whether SMURF1 regulates NRF2 in response to ER stress is poorly known. Thus, digging into the key mediator in glioblastoma cell survival upon ER stress could reveal the potential therapeutic target.

In this study, we identify overexpression of SMURF1 ameliorates ER stress by downregulating UPR pathway-mediated cell death and promoting ERAD activity. Moreover, SMURF1 targets KEAP1 for ubiquitination and degradation, leading to the activation of NRF2 signaling and reduction of ROS levels. Furthermore, SMURF1 promotes cell proliferation and growth in cooperation with NRF2. Taken together, our study highlights a crucial role of SMURF1 in maintaining ER homeostasis, and SMURF1 may be a potential target for glioblastoma therapy.

## Materials and methods

### Reagents and plasmids

Thapsigargin (TG) (Acros, 32875); Tunicamycin (TM) (Abcam, ab120296); Cycloheximide (CHX) (Merck, 239764); MG132 (MCE, HY-13259); Bafilimycin A1(Baf-A1) (MCE, HY-100558); Chloroquine (CQ) (Sigma, C6628); 4-phenylbutyric acid (4-PBA) (Target Mol, T5886); ISRIB (trans-isomer) (MCE, 1597403-47-8); N-acetyl-L-cysteine (NAC) (Adamas-beta, 616-91-1); tert-butylhydroquinone (tBHQ) (Adamas-beta, 1948-33-0); Lipofectamine^®^ RNAiMax reagent (Invitrogen, 13778150); Lipofectamine^®^ 2000 reagent (Invitrogen, 11668019); Reactive oxygen species assay kit (Beyotime, S0033S); Annexin V-FITC/PI detection kit (Solarbio, CA1020). Full-length SMURF1 cDNA was amplified from a human fetal brain cDNA library (Clonetech) and then inserted into 3×FLAG. Flag-SMURF1-C2, Flag-SMURF1-WW, Flag-SMURF1-ΔC2, and Flag-SMURF1-ΔHECT deletion constructs were created by subcloning polymerase chain reaction (PCR) products amplified. Flag-SMURF1-ΔWW deletion construct and Flag-SMURF1-C699A were generated by the site-directed mutagenesis using MutanBEST kit (Takara). HA-SMURF1 was generated by excising full-length SMURF1 cDNA from 3×Flag-SMURF1 and inserting it into the PKH3-3×HA. Flag-KEAP1(#28023), Myc-NRF2 (#21555) and CD3-δ-YFP (#11951) were purchased from Addgene. GFP-KEAP1 was generated by excising full-length KEAP1 cDNA from 3×Flag-KEAP1 and inserting it into the pEGFP-N3 (Clontech) vector. All constructs were confirmed by sequencing.

### Antibodies

Primary antibodies used against proteins were as follow: SMURF1 (Santa Cruz, sc-100616), SMURF1 (Abcam, ab57573), BiP/GRP78 (ABclonal, A0241); Actin (Sigma, A1978), p62 (MBL, PM045); LC3B (Sigma, L7543); Phospho-IRE1(S724) (ABclonal, AP0878); IRE1(ABclonal, A17940); Phospho-JNK1/2/3 (T183+T183+T221) (Abmart, T55541); JNK (ABclonal, A4867); Phospho-eIF2α (Ser51) (ABclonal, AP0692); eIF2α (ABclonal, A0764); XBP1 (ABclonal, A1731); ATF4 (Santa Cruz, sc-390063); CHOP (ABclonal, A6504); BCL-2 (ABmart, T40056); Flag M2 (Sigma, F3165); GFP-tag (Proteintech, 66002-1-lg); HA-tag (MBL, M180-3); Myc-Tag (Proteintech, 16286-1-AP); NRF2 (Proteintech, 16396-1-AP); KEAP1 (Proteintech, 10503-2-AP); Ubiquitin (MBL, D058-3); alpha Tubulin (Abcam, ab7291), Histone H2B (Santa Cruz, sc-515808); Caspase3 (Santa Cruz, sc-7272); Ki67 (Abcam, ab16667).

Secondary antibodies used were as follow: goat anti-mouse IgG secondary antibody (BOSTER, BA1050); goat anti-rabbit IgG secondary antibody (BOSTER, BA1054); Alexa Fluor^®^ 555 goat anti-mouse IgG (Life Technologies, A21425); ImmPRESS^TM^ HRP anti-Rabbit IgG (VECTOR, MP-7401); ImmPRESS^TM^ HRP anti-Mouse IgG (VECTOR, MP-7402); Rabbit anti-mouse IgG (CST, 58802) and Mouse anti-Rabbit IgG (CST, 93702) were used to avoid interference of the IgG heavy chain.

### Cell culture and transfection

The glioblastoma LN229 and U343 cell lines and Human Embryonic Kidney Epithelial cell 293A cells were cultured in Dulbecco’s Modified Eagle’s Medium (DMEM), supplemented with 10% fetal bovine serum (FBS) and 1% penicillin-streptomycin, at 37 °C with 5% CO_2_. The SMURF1-siRNA (5’-GCGUUUGGAUCUAUGCAAATT-3’, 3’-UUUGCAUAGAUCCAAACGCTT-5’) and NRF2-siRNA (GAAUGGUCCUAAAACACCATT) were purchased from JTSBIO (Wuhan, China). Cells were transfected with siRNA using Lipofectamine^®^ RNAiMax reagent and transfected with plasmids by Lipofectamine^®^ 2000 reagent, according to the manufacturer’s instructions.

### Western blotting

Cells were lysed in RIPA buffer (50 mM Tris, pH8.0; 150 mM NaCl; 1% NP40; 0.5% sodium deoxycholate; 0.1% SDS) supplemented with 1 mM PMSF and phosphatase inhibitor (BOSTER), and boiled with 5×loading buffer for 10 min. Protein samples were separated on 10-13.5% SDS-PAGE and transferred onto nitrocellulose membranes. Then, the membranes were blocked with 5% nonfat dry milk for 1 h at room temperature followed by incubating with primary antibody overnight at 4 °C. The membranes were washed with 0.1% Tween-20/TBS (TBST) and incubated with HRP-conjugated secondary antibody for 1 h at room temperature. After washing with 0.1% TBST, the bands on membranes were visualized using chemiluminescence (ECL) and analyzed using ImageJ.

### Quantitative real-time PCR

Total RNA was extracted from cells by using TRNzol Universal reagent (TIANGEN, DP424) according to manufacturer’s protocol. 1 μg RNA was used for reverse transcription by the Fast King RT Kit (With gDNase) (TIANGEN, KR116-02). Quantitative real-time (qRT) PCR was performed in Applied Biosystems 7500 using SYBR green PCR mix (ABclonal, RK21203) and the indicated primers (Supplementary Table 1).

### Cycloheximide chase assay

The cycloheximide (CHX) chase assay was performed to detect the degradation of proteins. Cells were treated with CHX (100 μg/mL), a protein synthesis inhibitor, for the indicated time and collected for western blot assay with the indicated antibodies.

### Immunoprecipitation

For immunoprecipitation, cells were lysed with lysis buffer (50 mM Tris, pH8.0; 150 mM NaCl; 1% NP40; 0.5% sodium deoxycholate) supplemented with 1 mM PMSF and phosphatase inhibitor for 30 min on ice, followed by sonication and centrifuging 12000 rpm for 15 min at 4 °C. The supernatant of lysates was collected and incubated with the primary antibody and protein G agarose beads (Solarbio, R8300) at 4 °C for 4 to 6 h. The beads were washed with ice-cold PBS, boiled in SDS sample buffer and identified by western blot assay.

### Immunofluorescence

Cells seeded on the coverslips were fixed with 4% paraformaldehyde for 15 min, permeabilized in 0.1% Triton X-100 for 5 min, followed by blocking with 1% BSA for 1 h at room temperature. Next, cells were incubated with primary antibody overnight at 4 °C, washed with 0.1% Tween-20/PBS (PBST), followed by incubating with Alexa Fluor-conjugated secondary antibody for 1 h at room temperature and staining the nucleus with DAPI dye. Cell images were visualized with confocal microscope (Nikon).

### Immunohistochemistry

Tissues were embedded in paraffin and mounted on slides. The slides were deparaffinized in xylene solution and rehydrated in ethanol. Antigen retrieval was performed in sodium citrate buffer by heating in microwave for 10 min and cooling at room temperature. The slides were permeabilized in 0.1% Triton X-100 for 5 min, blocked by 10% FBS for 1 h. Next, the slides were incubated with primary antibodies overnight at 4 °C, followed by washing with 0.1% PBST and incubating with ImmPRESS^TM^ HRP secondary antibody for 1 h at room temperature. The slides were washed and stained with ImmPACT^TM^ DAB (VECTOR, SK-4105), counterstained with hematoxylin, dehydrated through sequential ethanol grading, cleared in xylene and mounted. The slides image was observed by OLYMPUS SLIDEVIEW VS200.

### Cytoplasmic and nuclear fractionation

Cells were lysed using buffer (320 mM Sucrose; 3 mM CaCl_2_; 2 mM MgAc; 0.1 mM EDTA; 1 mM DTT; 0.5 mM PMSF) with 0.5% NP40 for 30 min on ice, followed by centrifuging at 600 *g* for 15 min at 4 °C. The supernatant was removed as cytoplasmic fraction, and the precipitate was nuclear fraction.

### Reactive oxygen species assay

The adherent cells were incubated in 1 mL DMEM medium with 1 μL fluorescence probe DCFH-DA and cultured at 37 °C for 20 min, followed by trypsin digestion. Cells were washed with DMEM medium and suspended in PBS. The cellular relative ROS levels were detected by FACS Calibur (BD) and analyzed by Flow Jo.

### Annexin-V/propidium iodide (PI) staining

Cells were collected and suspended in 500 μL 1×binding buffer supplemented with 5 μL Annexin-V fluorescein isothiocyanate and 5 μL propidium iodide (PI) for 15 min in the dark. The apoptotic cells were measured by FACS Calibur (BD) and analyzed by Flow Jo.

### Animal experiment

The animal experiments were performed according to a protocol that approved by the Institutional Animal Care and Use Committee of Beijing Institute of Technology. The shPLKO, shSMURF1 or shNRF2 LN229 cells (1 × 10^7^) in 100 μL PBS along with 20% Matrigel were injected into the left and right flanks of random eight-week-old female nude mice (*n* = 6). After 30 days of tumor growth, the tumor was removed and analyzed by western blot and Immunohistochemistry.

### Statistical analysis

Each experiment was repeated independently three times. The gray of western blot bands and fluorescence signals were analyzed by Image J. The statistical analysis of data was using the unpaired two-tailed Student’s *t-*test. *P* value less than 0.05 was considered significant and less than 0.01 was extremely significant. All data is shown as the mean ± SD. The graphical presentation was conducted using Microsoft Office Excel.

## Results

### ER stress induces the degradation of SMURF1

**Fig. 1:**
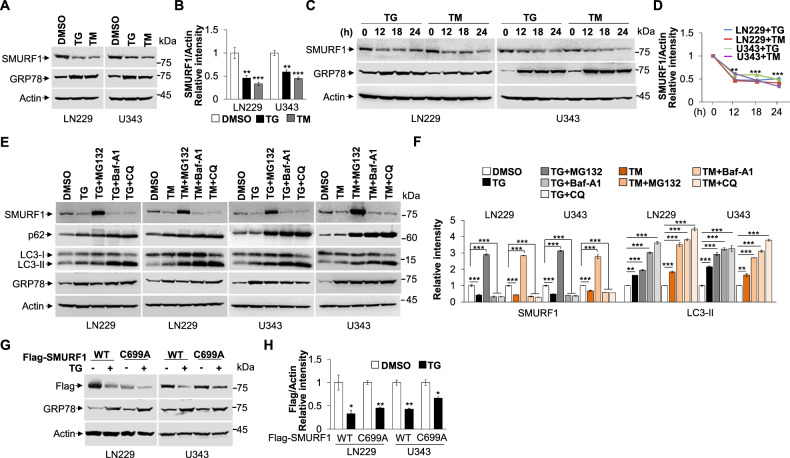
ER stress induces the degradation of SMURF1. **A** The LN229 and U343 cells were treated with or without Thapsigargin (TG, 1 μM) or Tunicamycin (TM, 10 μg/mL) for 12 h, and the whole cell extracts were analyzed by western blotting with antibodies against SMURF1, GRP78 and Actin. **B** Quantification of relative intensity of SMURF1 in (**A**). **C** The LN229 and U343 cells treated with TG (1 μM) or TM (10 μg/mL) were collected at the indicated time and analyzed by western blotting with antibodies against SMURF1, GRP78 and Actin. **D** Quantification of relative intensity of SMURF1 in (**C**). **E** The LN229 and U343 cells were treated with TG (1 μM) or TM (10 μg/mL) with or without proteasome inhibitor MG132 (10 μM) or autophagy inhibitor Bafilimycin A1 (Baf-A1, 100 nM) or autophagy inhibitor Chloroquine (CQ, 100 μM) for 12 h. The whole cell extracts were analyzed by western blotting with antibodies against SMURF1, p62, LC3B, GRP78 and Actin. **F** Quantification of relative intensity of SMURF1 and LC3-II in (**E**). **G** The LN229 and U343 cells were transfected with Flag-SMURF1 or E3 ligase-inactive mutant Flag-SMURF1-C699A plasmid for 24 h and treated with or without TG (1 μM) for 12 h. The whole cell extracts were analyzed by western blotting with antibodies against Flag, GRP78 and Actin. **H** Quantification of relative intensity of Flag-SMURF1 and Flag-SMURF1-C699A in (**G**). Data are presented as mean ± SD, (^*^p < 0.05, ^**^p < 0.01 and ^***^p < 0.001).

To investigate the role of SMURF1 in the ER stress, we initially detected the protein level of SMURF1 by the treatment of ER stress inducer TG and TM. Intriguingly, we found that the LN229 and U343 cells treated with low or high concentration of TG or TM for more than 12 h could induce significant downregulation of endogenous SMURF1 protein level (Fig. 1A–D and Fig. S1A, B). TG and TM successfully induced ER stress as indicated by the significant upregulation of ER chaperone BiP/GRP78, one of the ER stress markers. Importantly, ER stress-triggered downregulation of SMURF1 was blocked by proteasome inhibitor MG132 but not by autophagy inhibitor Bafilomycin A1 (Baf-A1) or Chloroquine (CQ), indicating that the degradation of SMURF1 protein upon ER stress depends on proteasome-mediated degradation system (Fig. 1E, F). Moreover, we found that the mRNA level of SMURF1 was not affected by TG or TM treatment (Fig. S1C). To explore the role of E3 ligase activity of SMURF1 in its degradation, we overexpressed Flag-SMURF1-C699A (SMURF1 mutant, loss of E3 ubiquitin activity) in the LN229 and U343 cells, and found that TG treatment also induced the degradation of Flag-SMURF1-C699A, indicating the ER stress-triggered downregulation of SMURF1 is independent of its E3 ligase activity (Fig. 1G, H and Fig. S1D). Taken together, these results suggest that ER stress promotes the proteasomal degradation of SMURF1.

### Knockdown of SMURF1 enhances UPR signaling-mediated cell death

**Fig. 2:**
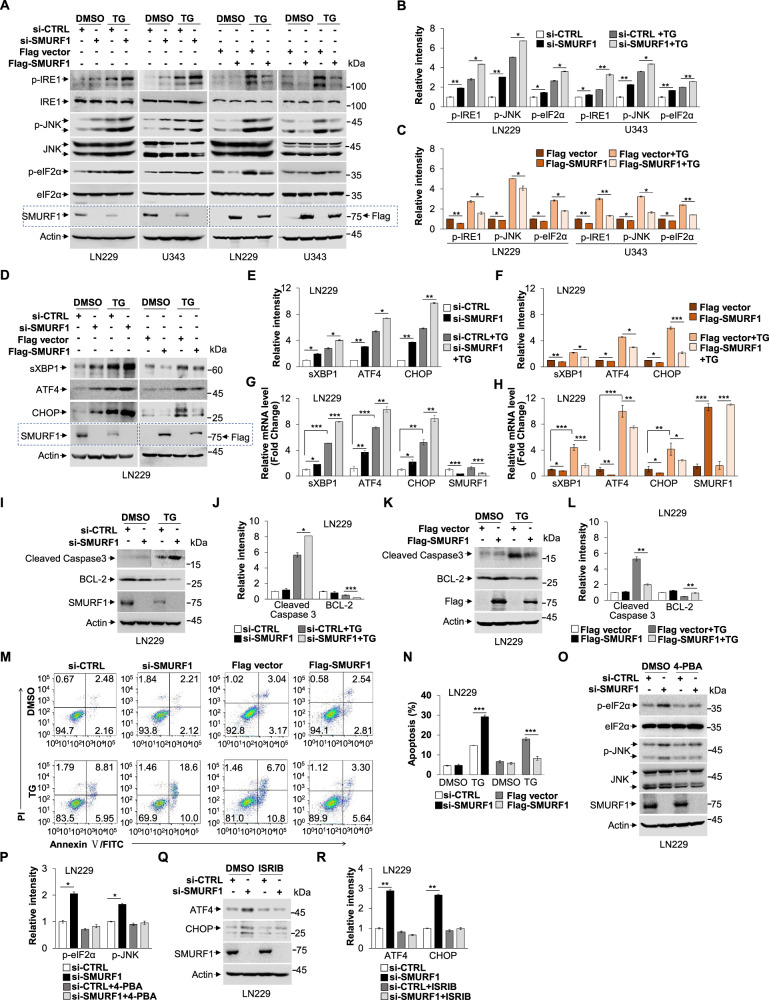
Knockdown of SMURF1 enhances UPR signaling mediated-cell death. **A** The LN229 and U343 cells were transfected with SMURF1 or scramble siRNA oligos for 72 h or transfected with Flag-SMURF1 or Flag vector plasmid for 24 h, and treated with or without TG (1 μM) for 12 h. The whole cell extracts were analyzed by western blotting with antibodies against phospho-IRE1/IRE1, phospho-JNK/JNK, phospho-eIF2α/eIF2α, SMURF1, Flag and Actin. **B**, **C** Quantification of relative intensity of phospho-IRE1/IRE1, phospho-JNK/JNK and phospho-eIF2α/eIF2α in (**A**). **D** The LN229 cells were transfected with SMURF1 or scramble siRNA oligos for 72 h or transfected with Flag-SMURF1 or Flag vector plasmid for 24 h, and treated with or without TG (1 μM) for 12 h. The whole cell extracts were analyzed by western blotting with antibodies against XBP1, ATF4, CHOP, SMURF1, Flag and Actin. **E**, **F** Quantification of relative intensity of sXBP1, ATF4 and CHOP in (**D**). **G**, **H** The LN229 cells were transfected with SMURF1 or scramble siRNA oligos for 72 h (**G**) or transfected with Flag-SMURF1 or Flag vector plasmid for 24 h (**H**), and treated with or without TG (1 μM) for 12 h. The relative mRNA levels of sXBP1, ATF4, CHOP and SMURF1 were conducted by qRT-PCR analysis. **I** The LN229 cells were transfected with SMURF1 or scramble siRNA oligos for 72 h and treated with or without TG (1 μM) for 16 h. The whole cell extracts were analyzed by western blotting with antibodies against Caspase3, BCL-2, SMURF1 and Actin. **J** Quantification of relative intensity of Cleaved Caspase3 and BCL-2 in (**I**). **K** The LN229 cells were transfected with Flag-SMURF1 or Flag vector plasmid for 24 h and treated with or without TG (1 μM) for 16 h. The whole cell extracts were analyzed by western blotting with antibodies against Caspase3, BCL-2, Flag and Actin. **L** Quantification of relative intensity of Cleaved Caspase3 and BCL-2 in (**K**). **M** The LN229 cells were transfected with SMURF1 or scramble siRNA oligos for 72 h or transfected with Flag-SMURF1 or Flag vector plasmid for 24 h, and treated with or without TG (1 μM) for 16 h, then stained with Annexin-V/ propidium iodide (PI). The apoptotic cells were analyzed by flow cytometry. **N** Quantification of apoptosis in (**M**). **O** The LN229 cells were transfected with SMURF1 or scramble siRNA oligos for 72 h and treated with or without 4-phenylbutyric acid (4-PBA) (10 μM) for 24 h. The whole cell extracts were analyzed by western blotting with antibodies against phospho-eIF2α/eIF2α, phospho-JNK/JNK, SMURF1 and Actin. **P** Quantification of relative intensity of phospho-eIF2α/eIF2α and phospho-JNK/JNK in (**O**). **Q** The LN229 cells were transfected with SMURF1 or scramble siRNA oligos for 72 h and treated with or without ISRIB (trans-isomer) (200 nM) for 12 h. The whole cell extracts were analyzed by western blotting with antibodies against ATF4, CHOP, SMURF1 and Actin. **R** Quantification of relative intensity of ATF4 and CHOP in (**Q**). Data are presented as mean ± SD, (^*^p < 0.05, ^**^p < 0.01 and ^***^p < 0.001).

To reveal the role of SMURF1 in the ER stress, we determined whether SMURF1 regulates UPR signaling. Interestingly, we found that knockdown of SMURF1 significantly increased, but overexpression of SMURF1 decreased the protein levels of phosphorylation of IRE1, JNK and eIF2α, especially during ER stress induction (Fig. 2A–C). These data indicate that SMURF1 negatively regulates UPR signaling pathways. Moreover, we detected that SMURF1 depletion significantly increased, but overexpression of SMURF1 reduced the protein levels of spliced X-box-binding protein 1 (sXBP1), ATF4 and CHOP in the absence or presence of ER stress (Fig. 2D–F). Consistently, we observed that the mRNA levels of sXBP1, ATF4 and CHOP were significantly upregulated in the SMURF1 depleted cells but downregulated in the SMURF1 overexpressed cells compared to control cells with or without ER stress (Fig. 2G, H and Fig. S2A, B). These data further confirmed that SMURF1 was a crucial regulator upon ER stress by negatively controlling UPR. Of note, knocking down SMURF1 significantly increase CHOP, a major transcription factor that regulates ER stress-induced apoptosis, suggesting that SMURF1 contributes to protecting from UPR-mediated cell death. Indeed, we found that knockdown of SMURF1 significantly increased the level of Cleaved Caspase3 (apoptosis-related marker) but reduced the level of BCL-2 (anti-apoptosis marker) during ER stress (Fig. 2I, J). While overexpression of SMURF1 obviously reduced the level of Cleaved Caspase3 but increased the level of BCL-2 upon ER stress (Fig. 2K, L). Consistently, we also found that SMURF1 depletion significantly increased, but overexpression of SMURF1 significantly decreased cell apoptosis compared to control cells in presence of ER stress (Fig. 2M, N). These data confirmed that SMURF1 inhibits ER stress-triggered cell death. To further verify that SMURF1 knockdown activates UPR signaling and leads to cell death, we treated LN229 cells with or without chemical chaperones 4-phenylbutyric acid (4-PBA) that is known to improve ER folding capacity and alleviate ER stress, and UPR (PERK) inhibitor ISRIB (trans-isomer). We found that 4-PBA treatment reversed the SMURF1 knockdown-induced the upregulation of phosphorylation of JNK and eIF2α (Fig. 2O, P). ISRIB treatment reversed the SMURF1 knockdown-induced increase of ATF4 and CHOP (Fig. 2Q, R). Altogether, these findings suggest knocking down SMURF1 exacerbates ER stress by upregulating UPR-mediated pro-death.

### SMURF1 knockdown enhances ROS production and impairs ERAD activity

**Fig. 3:**
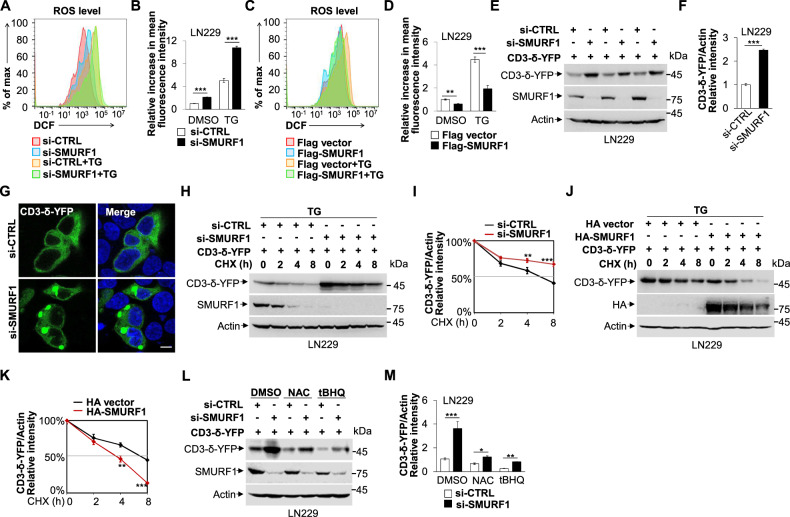
SMURF1 knockdown enhances ROS production and impairs ERAD activity. **A** The LN229 cells were transfected with SMURF1 or scramble siRNA oligos for 72 h and treated with or without TG (1 μM) for 12 h. Cells were then stained with 2,7-Dichlorodihydrofluorescein diacetate (DCFH-DA, 10 μM) and the ROS level was detected by flow cytometry. **B** Quantification of relative increase in mean fluorescence intensity in (**A**). **C** The LN229 cells were transfected with Flag-SMURF1 or Flag vector plasmid for 24 h and treated with or without TG (1 μM) for 12 h, then stained with DCFH-DA (10 μM), and the ROS level was detected by flow cytometry. **D** Quantification of relative increase in mean fluorescence intensity in (**C**). **E** The LN229 cells were transfected with CD3-δ-YFP plasmid for 12 h, and then transfected with SMURF1 or scramble siRNA oligos for 72 h. The whole cell extracts were analyzed by western blotting with antibodies against GFP, SMURF1 and Actin. **F** Quantification of relative intensity of CD3-δ-YFP in (**E**). **G** The 293A cells were transfected with CD3-δ-YFP plasmid for 12 h, and then transfected with SMURF1 or scramble siRNA oligos for 72 h and processed for immunofluorescence analysis. The nucleus was stained by DAPI (blue). Scale bar: 10 μm. YFP Excitation max (nm) 514 and Emission max (nm) 527. **H** The LN229 cells were transfected with CD3-δ-YFP plasmid for 12 h, and then transfected with SMURF1 or scramble siRNA oligos for 60 h. The LN229 cells were per-treated with TG (1 μM, 6 h) and then treated with cycloheximide (CHX, 100 μg/mL) for the indicated time. The whole cell extracts were analyzed by western blotting with antibodies against GFP, SMURF1 and Actin. **I** Quantification of CD3-δ-YFP band intensities relative to Actin. **J** The LN229 cells were transfected with CD3-δ-YFP plasmid for 12 h, and then transfected with HA-SMURF1 or HA vector plasmid for 24 h, per-treated with TG (1 μM, 6 h), followed by treatment with CHX (100 μg/ml). Cells were collected at the indicated time and analyzed by western blotting with antibodies against GFP, HA and Actin. **K** Quantification of CD3-δ-YFP band intensities relative to Actin. **L** The LN229 cells were transfected with CD3-δ-YFP plasmid for 12 h, and then transfected with SMURF1 or scramble siRNA oligos for 60 h, and treated with DMSO, NAC (2 mM) or tBHQ (20 μM) for 8 h. The whole cell extracts were analyzed by western blotting with antibodies against GFP, SMURF1 and Actin. **M** Quantification of CD3-δ-YFP band intensities relative to Actin. Data are presented as mean ± SD, (^*^p < 0.05, ^**^p < 0.01 and ^***^p < 0.001).

Previous reports have shown ROS overproduction is sufficient to disrupt protein folding and leads to ER stress [45, 46]. We then explored whether SMURF1 is feedback involved in the regulation of ROS production. We found that knockdown of SMURF1 significantly increased, but overexpression of SMURF1 attenuated the ROS level, suggesting SMURF1 negatively regulates ROS production (Fig. 3A–D). Studies have demonstrated that consistent accumulation of ROS mediated by UPR leads to activation quality-control machinery ERAD, which is responsible for the clearance of misfolded proteins for proteasomal degradation [47]. Next, we explored whether SMURF1 contributes to the clearance of misfolded proteins through ERAD. We found that the classic ERAD substrate CD3-δ-YFP was significantly accumulated in the LN229 cells with SMURF1 knockdown, suggesting ERAD activity is impaired in the absence of SMURF1 (Fig. 3E–G and Fig. S3A). Moreover, the depletion and overexpression of SMURF1 treated with protein synthesis inhibitor Cycloheximide (CHX) showed that SMURF1 facilitated the degradation of CD3-δ-YFP during ER stress in time dependent manner (Fig. 3H–K and Fig. S3B–E). These data suggest that SMURF1 promotes ERAD activity. We next determined whether SMURF1 regulated ROS could result in ERAD. To be noted, we treated SMURF1 depleted cells expressing CD3-δ-YFP with ROS scavenger N-acetyl-L-cysteine (NAC) or antioxidant NRF2 activator tert-butylhydroquinone (tBHQ) or 4-PBA, and found that the decreased ERAD was partially rescued in SMURF1 depletion cells, indicating SMURF1 promotes ERAD at least partially by controlling ROS levels (Fig. 3L, M and Fig. S3F–H). In addition, we also found that NAC treatment rescued SMURF1 knockdown-induced increase the levels of phosphorylation of IRE1/IRE1 and sXBP1 (Fig. S3I, J). Taken together, these data suggest that SMURF1 modulates ROS redox balance and ERAD activity upon ER stress.

### SMURF1 activates NRF2 signaling pathway by promoting its nuclear import

**Fig. 4:**
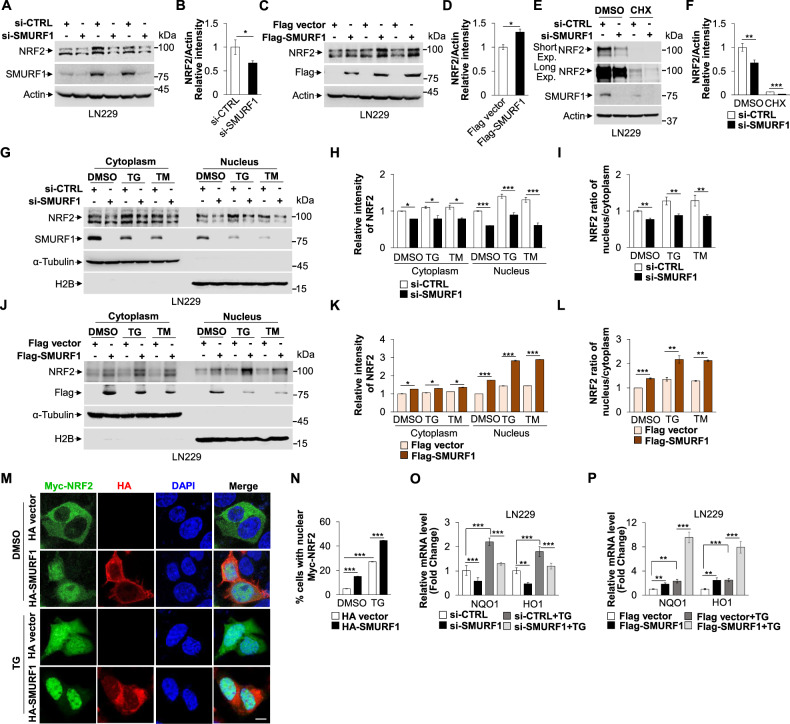
SMURF1 activates NRF2 signaling pathway by promoting its nuclear import. **A** The LN229 cells were transfected with SMURF1 or scramble siRNA oligos for 72 h and the whole cell extracts were analyzed by western blotting with antibodies against NRF2, SMURF1 and Actin. **B** Quantification of relative intensity of NRF2 in (**A**). **C** The LN229 cells were transfected with Flag-SMURF1 or Flag vector plasmid for 24 h and the whole cell extracts were analyzed by western blotting with antibodies against NRF2, Flag and Actin. **D** Quantification of relative intensity of NRF2 in (**C**). **E** The LN229 cells were transfected with SMURF1 or scramble siRNA oligos for 60 h and treated with DMSO, CHX (100 μg/mL) for 12 h. The whole cell extracts were analyzed by western blotting with antibodies against NRF2, SMURF1 and Actin. **F** Quantification of relative intensity of NRF2 in (**E**). **G** The LN229 cells transfected with SMURF1 or scramble siRNA oligos were treated with DMSO, TG (1 μM, 12 h) or TM (10 μg/ml, 12 h) and collected for the cytoplasmic and nuclear fractionation assay. The levels of NRF2, SMURF1, α-Tubulin and H2B were analyzed by western blotting. **H** Quantification of relative intensity of NRF2 in (**G**). **I** Quantification of NRF2 ratio of nucleus vs cytoplasm in (**G**). **J** The LN229 cells transfected with Flag-SMURF1 or Flag vector were treated with DMSO, TG (1 μM, 12 h) or TM (10 μg/mL, 12 h), and the cytoplasmic and nuclear fractionation of NRF2, Flag, α-Tubulin and H2B were measured by western blotting. **K** Quantification of relative intensity of NRF2 in (**J**). **L** Quantification of NRF2 ratio of nucleus vs cytoplasm in (**J**). **M** The 293A cells with Myc-NRF2 expression were transfected with HA vector or HA-SMURF1 plasmid and treated with or without TG (1 μM, 12 h). The localization of Myc-NRF2 in cells was performed by immunofluorescence analysis. The nucleus was stained by DAPI (blue). Scale bar: 10 μm. **N** Quantification of percent of cells with nuclear Myc-NRF2 in (**M**). **O** The LN229 cells transfected with SMURF1 or scramble siRNA oligos were treated with TG (1 μM, 12 h), and the relative mRNA levels of NQO1 and HO1 were conducted by qRT-PCR analysis. **P** The LN229 cells transfected with Flag-SMURF1 or Flag vector were treated with or without TG (1 μM, 12 h), and the relative mRNA levels of NQO1 and HO1 were performed by qRT-PCR analysis. Short Exp., short exposure; Long Exp., long exposure. Data are presented as mean ± SD, (^*^p < 0.05, ^**^p < 0.01 and ^***^p < 0.001).

NRF2 is the key transcriptional factor that regulates numerous antioxidant genes to mediate cellular stress response. Next, we tried to investigate the key role of NRF2 in SMURF1-mediated ER stress response. Surprisingly, we found that depletion of SMURF1 significantly downregulated, but overexpression of SMURF1 increased the protein level of NRF2 in the LN229 cells (Fig. 4A–D). Importantly, we found that SMURF1 suppressed NRF2 degradation, evidenced by depletion of SMURF1 with CHX treatment induced an increased degradation of NRF2 protein (Fig. 4E, F). Moreover, we found that the mRNA level of NRF2 was not affected with SMURF1 knockdown (Fig. S4A). Next, we explored whether SMURF1 regulated NRF2 nuclear translocation and transcriptional activity. We observed that knockdown of SMURF1 significantly suppressed, but overexpression of SMURF1 promoted the NRF2 nuclear import upon ER stress (Fig. 4G–L). Consistently, the immunofluorescent staining showed that overexpression of SMURF1 significantly increased nuclear localization of NRF2 compared to control cells in the absence or presence of ER stress (Fig. 4M, N). These data indicate that SMURF1 promotes NRF2 nuclear translocation in response to ER stress. Furthermore, we also verified that under TG or TM treatment, the mRNA levels of NRF2 targets (NQO1 and HO1) were significantly decreased in SMURF1 knockdown cells, but increased in SMURF1 overexpression cells compared to control cells (Fig. 4O, P and Fig. S4B, C). Altogether, these findings show that SMURF1 activates NRF2 signaling by promoting its nuclear import during ER stress.

### SMURF1 mediates the ubiquitination and degradation of KEAP1

**Fig. 5:**
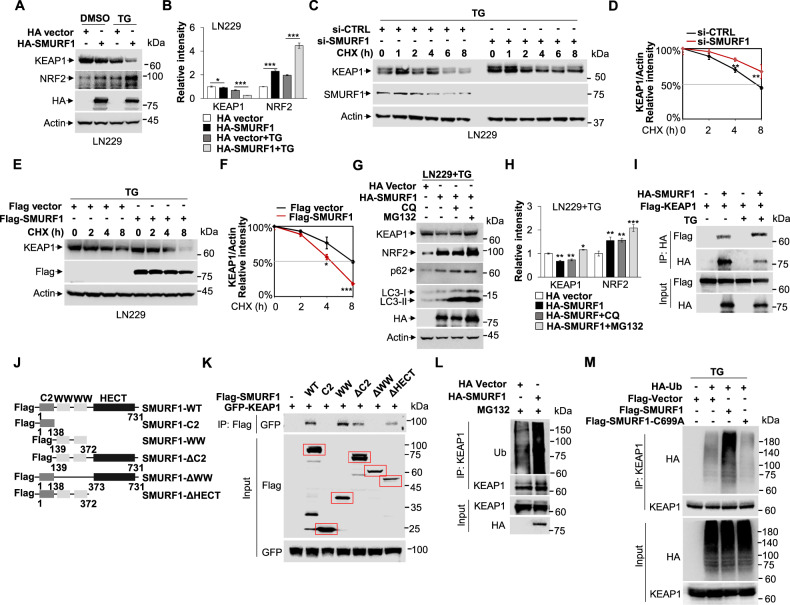
SMURF1 mediates the ubiquitination and degradation of KEAP1. **A** The LN229 cells transfected with HA vector or HA-SMURF1 were treated with or without TG (1 μM) for 12 h, and the whole cell extracts were analyzed by western blotting with antibodies against KEAP1, NRF2, HA and Actin. **B** Quantification of relative intensity of KEAP1 and NRF2 in (**A**). **C** The LN229 cells transfected with SMURF1 or scramble siRNA oligos were per-treated with TG (1 μM, 6 h), and then treated with CHX (100 μg/mL). Cells were collected at the indicated time and analyzed by western blotting with antibodies against KEAP1, SMURF1 and Actin. **D** Quantification of KEAP1 band intensities relative to Actin. **E** The LN229 cells transfected with Flag vector or Flag-SMURF1 were per-treated with TG (1 μM, 6 h), and then treated with CHX (100 μg/ml). Cells were collected at the indicated time and analyzed by western blotting with antibodies against KEAP1, Flag and Actin. **F** Quantification of KEAP1 band intensities relative to Actin. **G** The LN229 cells transfected with HA vector or HA-SMURF1 were treated with TG (1 μM) simultaneously with or without CQ (100 μM) or MG132 (10 μM) for 12 h. The whole cell extracts were analyzed by western blotting with antibodies against KEAP1, NRF2, p62, LC3B, HA and Actin. **H** Quantification of relative intensity of KEAP1 and NRF2 in (**G**). **I** The co-immunoprecipitation (co-IP) analysis of the interaction between HA-SMURF1 and Flag-KEAP1 in the LN229 cells with or without TG (1 μM, 12 h) treatment. **J** The presentation of domains of Flag-tagged SMURF1 and deletion constructs. **K** The LN229 cells overexpressing GFP-KEAP1 were transfected with Flag-SMURF1 and its deletion constructs, the key domain for interaction was detected by immunoprecipitation with Flag antibody and western blotting with antibodies against GFP and Flag. **L** The LN229 cells transfected with HA vector or HA-SMURF1 were treated with MG132 (10 μM, 16 h). The ubiquitination of KEAP1 was analyzed by immunoprecipitation with KEAP1 antibody and western blotting with antibodies against Ubiquitin, KEAP1 and HA. **M** The LN229 cells transfected with Flag-vector, Flag-SMURF1 or Flag-SMURF1-C699A were transfected with HA-Ub and treated with TG (1 μM, 12 h). The ubiquitination of KEAP1 was analyzed by immunoprecipitation with KEAP1 antibody and western blotting with antibodies against HA and KEAP1. Data are presented as mean ± SD, (^*^p < 0.05, ^**^p < 0.01 and ^***^p < 0.001).

Next, we asked whether the SMURF1-mediated NRF2 nuclear import is associated with KEAP1 degradation. Intriguingly, knockdown of SMURF1 significantly increased the protein level of KEAP1 (Fig. S5A, B). In contrast, overexpression of SMURF1 significantly decreased KEAP1 level under basal and ER stress conditions (Fig. 5A, B and Fig. S5C, D). Of note, knockdown of SMURF1 did not affect the mRNA level of KEAP1 (Fig. S5E). Moreover, both SMURF1 depletion and overexpression with CHX treatment experiments showed that SMURF1 affected KEAP1 protein level by reducing its stability (Fig. 5C–F). Furthermore, ER stress-triggered KEAP1 protein degradation was significantly blocked by proteasome inhibitor MG132, but not by autophagy inhibitor CQ, indicating that SMURF1 promotes the degradation of KEAP1 through the ubiquitin-proteasome pathway during ER stress (Fig. 5G, H). To investigate whether KEAP1 is the potential substrate of SMURF1, we performed co-immunoprecipitation (co-IP) to identify their interaction. The results showed that the interaction between SMURF1 and KEAP1 was significantly increased during ER stress compared with basal condition, suggesting the regulation of SMURF1 on KEAP1 and/or NRF2 nuclear import is more obvious under ER stress condition even though the downregulation of SMURF1 (Fig. 5I and Fig. S5F). To further investigate which domain of SMURF1 is responsible for the interaction with KEAP1, we constructed different SMURF1 deletion mutants (Fig. 5J). Importantly, full-length construct, WW, ΔC2 and ΔHECT domains of SMURF1 were able to, but the ΔWW domain and C2 domain were not able to interact with GFP-KEAP1, suggesting WW domain is required for the SMURF1-KEAP1 interaction (Fig. 5K). We hypothesized that E3 ligase SMURF1 mediates the ubiquitination and degradation of KEAP1. Expectedly, we found Flag-SMURF1, but not Flag-SMURF1-C699A, increased ubiquitination level of KEAP1, suggesting the E3 ligase activity is indispensable for SMURF1 to promote the ubiquitination of KEAP1 under ER stress (Fig. 5L, M). Moreover, we detected that the E3 ligase activity was required for SMURF1 to mediate NRF2 nuclear translocation and transcriptional activity (Fig. S5G–I). Taken together, these findings show the novel regulatory mechanism of NRF2 signaling pathway that SMURF1 mediates KEAP1 degradation through its ubiquitination modification.

### SMURF1 protects cell survival in NRF2 dependent manner

**Fig. 6:**
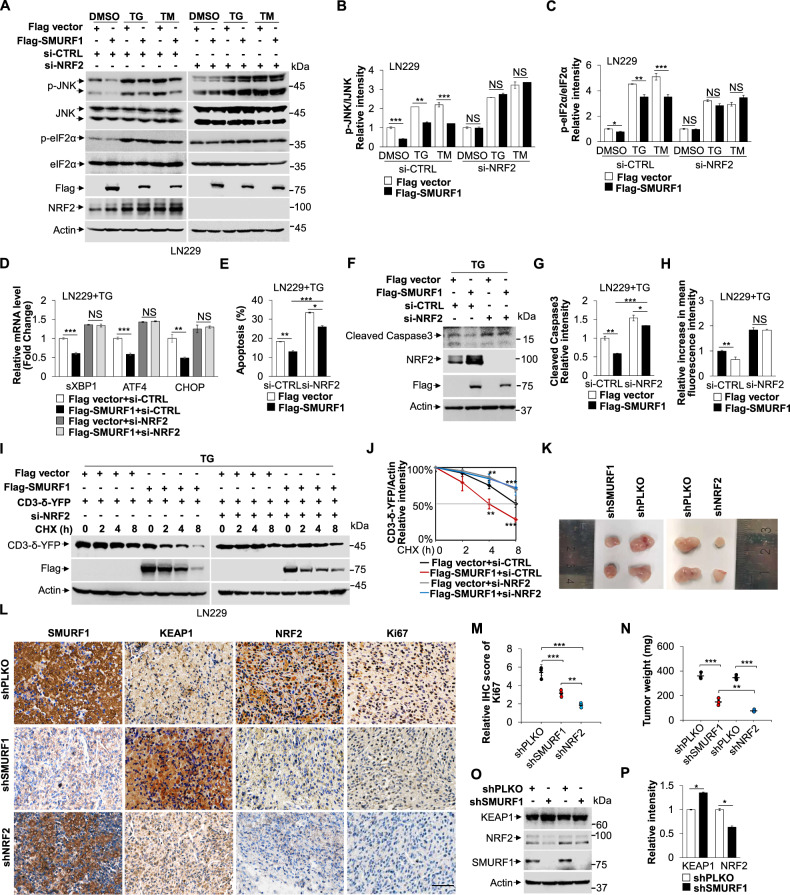
SMURF1 protects cell survival in a NRF2 dependent manner. **A** The LN229 cells were transfected with NRF2 or scramble siRNA oligos for 48 h and transfected with Flag vector or Flag-SMURF1 plasmid for 24 h, then treated with or without TG (1 μM, 12 h) or TM (10 μg/mL, 12 h). The whole cell extracts were analyzed by western blotting with antibodies against phospho-JNK/JNK, phospho-eIF2α/eIF2α, Flag, NRF2 and Actin. **B** Quantification of relative intensity of phospho-JNK/JNK in (**A**). **C** Quantification of relative intensity of phospho-eIF2α/eIF2α in (**A**). **D** The LN229 cells were transfected with NRF2 or scramble siRNA oligos for 48 h and transfected with Flag vector or Flag-SMURF1 plasmid for 24 h and treated with TG (1 μM, 12 h). The relative mRNA levels of sXBP1, ATF4 and CHOP were performed by qRT-PCR analysis. **E** The LN229 cells were transfected with NRF2 or scramble siRNA oligos for 48 h and transfected with Flag vector or Flag-SMURF1 plasmid for 24 h and treated with TG (1 μM, 16 h). Cells were stained with Annexin-V/PI and analyzed by flow cytometry. The graph represented the quantification of apoptosis. **F** The LN229 cells were transfected with NRF2 or scramble siRNA oligos for 48 h and transfected with Flag vector or Flag-SMURF1 plasmid for 24 h and treated with TG (1 μM, 16 h). The whole cell extracts were analyzed by western blotting with antibodies against Caspase3, NRF2, Flag and Actin. **G** Quantification of relative intensity of Cleaved Caspase3. **H** The LN229 cells were transfected with NRF2 or scramble siRNA oligos for 48 h and transfected with Flag vector or Flag-SMURF1 plasmid for 24 h and treated with TG (1 μM, 12 h). Cells were then stained with DCFH-DA (10 μM) and the ROS levels were detected by flow cytometry. The graph showed the quantified data of relative increase in mean fluorescence intensity. **I** The LN229 cells were transfected with NRF2 or scramble siRNA oligos for 48 h and transfected with Flag vector or Flag-SMURF1 plasmid for 24 h and pre-treated with TG (1 μM, 6 h), then treated with CHX (100 μg/mL). Cells were collected at the indicated time and analyzed by western blotting with antibodies against GFP, Flag and Actin. **J** The quantification of CD3-δ-YFP band intensities relative to Actin. **K** The LN229 cells with shControl, shSMURF1 or shNRF2 were subcutaneously injected in the right or left side of nude mice (*n* = 6), respectively. The representative image of tumor size was showed at day 30. **L** Immunohistochemistry analysis of tumor tissue slides with antibodies against SMURF1, KEAP1, NRF2 and Ki67. Nucleus was stained by hematoxylin. Scale bar, 50 μm. **M** The graph showed the quantified data of Ki67. **N** The graph showed the quantified data of tumor weight. **O** Western blot analysis of tumor tissue lysates from two different mice with antibodies against KEAP1, NRF2, SMURF1 and Actin. **P** Quantification of relative intensity of KEAP1 and NRF2 in (**O**). Data are presented as mean ± SD, (^*^p < 0.05, ^**^p < 0.01 and ^***^p < 0.001).

**Fig. 7:**
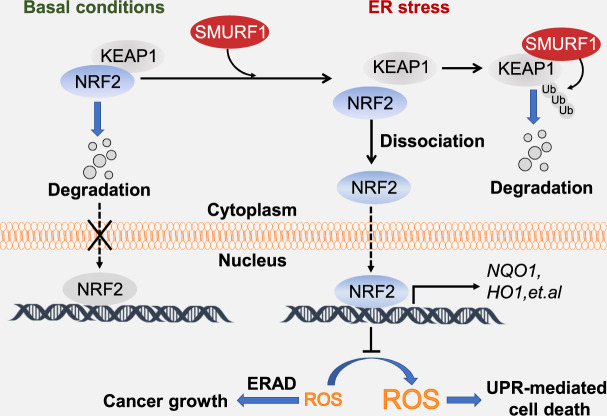
SMURF1 promotes tumor cell survival from ER stress. A schematic model illustrating that SMURF1 regulates KEAP1-NRF2 signaling pathway upon ER stress to promote glioblastoma survival. At basal conditions, KEAP1 binds to and sequesters NRF2 in the cytoplasm, resulting in proteasome degradation of NRF2. During ER stress, SMURF1 targets KEAP1 for ubiquitination and degradation, leading to translocation of NRF2 into the nucleus, where NRF2 further activates its downstream antioxidant genes (NQO1 and HO1, et al.) to reduce ROS levels, thereby promoting cancer growth through ERAD activity and inhibiting UPR-mediated cell death.

Next, we assessed whether the protective role of SMURF1 depends on NRF2 in response to ER stress. Indeed, depletion of NRF2 suppressed SMURF1 overexpression-mediated the decrease of UPR signaling (Fig. 6A–D). Moreover, the protecting role of SMURF1 for cancer cell survival was also impaired with the NRF2 depletion in LN229 cells (Fig. 6E–G and Fig. S6A, B). Furthermore, overexpression of SMURF1 failed to reduce ROS levels and activate ERAD by knockdown of NRF2 (Fig. 6H–J and Fig. S6C). These results indicate that NRF2 is required for SMURF1 mediated ER stress response. To investigate the role of SMURF1 in vivo and compare the effect among shSMURF1 and shNRF2, we explanted LN229 cells with control, SMURF1 or NRF2 depletion in nude mice. The tumor size of the SMURF1 or NRF2 deletion groups was smaller than that of the control group, but the tumor size of the NRF2 deletion group was smaller than that of the SMURF1 deletion group (Fig. 6K, N). Moreover, the cell proliferation marker Ki67 was significantly decreased in the SMURF1 and NRF2-deficient tumor compared with the control group, and the Ki67 was significantly decreased in the NRF2-deficient tumor compared with the SMURF1-deficient tumor cells (Fig. 6L, M). These data indicate that NRF2 is required for cell growth and proliferation. Furthermore, SMURF1 depleted tumor showed downregulation of NRF2 expression, and upregulation of KEAP1 expression compared to the control tumor xenografts, suggesting that SMURF1-induced KEAP1-NRF2 pathway plays a vital role in glioblastoma growth (Fig. 6L, O, P). Altogether, our findings indicated that SMURF1 targets KEAP1 for its ubiquitination and degradation, leading to translocation of NRF2 into the nucleus, where NRF2 further activates its downstream antioxidant genes to reduce ROS levels, thereby preventing ER-induced cell death (Fig. 7).

## Discussion

To cope with the unfolded and/or misfolded proteins and restore ER function, tumor cells evoke an ER stress management of integrated signaling system, including the UPR pathway and ERAD to facilitate the protein folding and/or degradation [47, 48]. Whereas, unresolved and extreme ER stress induces excessive ROS accumulation, which is followed by oxidative stress-mediated cell death. The KEAP1-NRF2 pathway is well-established as the crucial antioxidant signaling to suppress ER stress-induced apoptosis by eliminating ROS and inducing pro-survival gene expression [19, 20]. Here, we identified that SMURF1 attenuates ER stress by negatively regulating UPR signaling, promoting ERAD activity and reducing ROS levels. Mechanistically, SMURF1 targets KEAP1 by ubiquitination and degradation, resulting in activation of NRF2 signaling. Therefore, our findings suggest that SMURF1 is a novel regulator in ER stress and plays a cytoprotective role in glioblastoma.

The highly expressed SMURF1 in glioblastoma cells is required for ER homeostasis maintenance. Our study reveals the details of SMURF1 in defending against ER stress. Importantly, SMURF1 is a crucial regulator for negatively controlling UPR signaling. Generally, the adaptive UPR fails to preserve ER homeostasis and terminal UPR is engaged, leading to cell apoptosis [49]. For instance, ER stress-induced JNK activation is thought to initiate a pro-apoptotic pathway by promoting de novo synthesis of death receptors and their ligands and by targeting components of the BCL-2 family [48, 50]. In addition, the activation of PERK/eIF2α/ATF4/CHOP axis suppresses tumor progression and triggers cell death [18, 51]. We found that although the IRE1 and PERK signaling in the UPR pathway of ER stress response were activated in SMURF1 knockdown cells, the phosphorylation of JNK and eIF2α failed to produce such a beneficial function for cell survival, but led to apoptosis, suggesting activation of severe ER stress in the absence of SMURF1. Moreover, the protein levels of pro-apoptotic CHOP and Cleaved Caspase3 were significantly increased, but anti-apoptotic BCL-2 was significantly decreased in SMURF1 knockdown cells compared to control cells, indicating UPR-mediated apoptosis was induced in the absence of SMURF1. In addition, blocking the activation of PERK pathway in SMURF1 depleted cells with ISRIB could impair the increased effects of SMURF1 depletion on CHOP expression, supporting a role of CHOP in SMURF1 depletion-mediated cell death. Furthermore, other studies have reported that ERAD-deficient HepG2 cells present enhanced ROS accumulation [52]. We also found that impaired ERAD activity was in the absence of SMURF1. Given that removing ROS rescues the decreased ERAD, we consider that SMURF1 positively regulates ERAD by partially controlling ROS levels. Altogether, our study suggests that depletion of SMURF1 triggers ER stress-induced apoptosis. Our study also emphasizes SMURF1 activates the KEAP1-NRF2 signaling, which contributes to SMURF1-mediated cytoprotective function. Previous studies have reported that ER stress activates NRF2 for antioxidant defense and cell survival [21]. Consistently, we found that TG or TM treatment induced NRF2 activation, thus the expression of NRF2 target genes (HO1 and NQO1) were increased during ER stress. The KEAP1-NRF2 signaling can be activated by 1) KEAP1 disassociation from NRF2; 2) NRF2 phosphorylation regulation [27]; 3) KEAP1 degradation [23, 24], etc. Recent studies have revealed that TRIM25 as a RING-type E3 ubiquitin ligase facilitates tumor cell survival by activating NRF2 signaling through ubiquitination and degradation of KEAP1 during ER stress, providing a promising therapeutic approach targeting TRIM25 concurrently with NRF2 inhibition [53]. Our study defines a novel mechanism that SMURF1 activates NRF2 signaling by ubiquitination and degradation of KEAP1. We suggest that overexpression of SMURF1 promotes NRF2 nuclear import and activates its target genes (HO1 and NQO1) expression, resulting in reduced ROS levels. Interestingly, TG or TM induced Smurf1 degradation through UPS system, thus may fail to activate NRF2. However, the SMURF1 significantly increased Nrf2 protein level and decreased KEAP1 protein level upon TG treatment. Importantly, we noted that SMURF1 increased the interaction with and ubiquitination of KEAP1 for its degradation upon ER stress, indicating the activation of NRF2 signaling is more significant during ER stress. Moreover, this regulation is dependent on SMURF1 E3 ubiquitin activity, which is supported by the failure of the ubiquitin ligase-defective mutant, Flag-SMURF1-C699A, to promote NRF2 nuclear import and NRF2 transcriptional activity. Previous studies have shown that NRF2 is the direct PERK substrate and a critical effector of PERK-mediated cell survival [27]. Our work reveals that overexpression of SMURF1 suppresses PERK signaling but increases NRF2 expression, indicating that the activation of NRF2 signaling by SMURF1 is independent of the PERK pathway. Finally, the mouse xenograft models also confirmed that depletion of SMURF1 markedly suppressed glioblastoma cell growth and proliferation, which was associated with decreased KEAP1-NRF2 pathway. Taken together, our studies identify that SMURF1 involves in ER stress management by modulating KEAP1-NRF2 pathway.

Our study suggested that SMURF1 expression conferred cells resistance to ER stress inducers that also caused SMURF1 ubiquitination and degradation. Generally, many crucial regulators are in response to ER stress by induced expression. For instance, pharmacologic ER stress agents induce pro-oncogene SEC61γ expression, which in turn confers growth advantage in glioblastoma cells [54]. However, previous studies have reported that ER stress inducers cause FK506-binding protein 9 (FKBP9) degradation in a proteasomal-dependent manner but FKBP9 expression confers resistance to ER stress inducer-triggered cell death [53]. Therefore, the degraded protein can also be crucial regulator upon ER stress. But what factor causes SMURF1 degradation upon ER stress is unclear and needs further study. Indeed, ER stress has been described as an inducer of autophagy, we also detected that LC3II level was significantly increased after TG or TM treatment. Moreover, previous studies have shown that ER stress could induce reticulophagy, which in turn restores cellular energy levels and ER homeostasis [55]. Recent studies have shown that the transcription factor ATF4, which is accompanied by the induction of additional ER stress markers, links ER stress with reticulophagy in glioblastoma cells [56]. Flavokawain B (FKB), a natural kava chalcone, inhibits glioblastoma growth through inducing ER stress induced autophagy [57]. Since SMURF1 is involved in selective autophagy, we suspected that the reduction of SMURF1 is due to ER stress-induced autophagy activation. However, we found that the downregulation of SMURF1 was blocked by proteasome inhibitor MG132 but not by autophagy inhibitor Baf-A1 or CQ, suggesting the degradation of SMURF1 protein upon ER stress depends on proteasome-mediated degradation system. Therefore, which the E3 ligase is involved in SMURF1 degradation upon ER stress needs further study.

Studies have demonstrated that ER stress-inducing drugs, either as a monotherapy or in conjunction with TMZ and radiation, could be a promising therapeutic strategy for glioblastoma [58]. Understanding the molecular mechanisms of tumor cells defending against ER stress will provide newer potential therapeutic targets for developing efficient therapies. Our work provides a novel mechanism that SMURF1 defends against ER stress and promotes glioblastoma cell survival through KEAP1-NRF2 pathway. Therefore, targeting SMURF1 and KEAP1-NRF2 signaling pathway may be potential cancer therapy for glioblastoma or ER stress-related disease. Further studies are needed to explore the molecular mechanisms of glioblastoma through which ER stress induces the degradation of SMURF1, and how to mediate its pro-survival effects and whether there is a feedback regulation mechanism affecting SMURF1. Understanding of molecular mechanism of SMURF1 involved in ER stress response will provide the insight of SMURF1 as a potential target for glioblastoma therapy.

## Supplementary information

Table 1

Supplementary

checklist

Co-authors email responses

Supplementary Figure 1

Supplementary Figure 2

Supplementary Figure 3

Supplementary Figure 4

Supplementary Figure 5

Supplementary Figure 6

Original Data File

## Data Availability

The datasets generated during the current study are available from the corresponding author on reasonable request.
