# Supplementary material for: SMURF1 attenuates endoplasmic reticulum stress by promoting the degradation of KEAP1 to activate NRF2 antioxidant pathway
Source: Cell Death Dis. 2023 Jun 14;14(6):361. doi: 10.1038/s41419-023-05873-2 (PMC10267134; doi:10.1038/s41419-023-05873-2)
Supplement: Supplementary file 1 — Table 1 [file 41419_2023_5873_MOESM1_ESM.docx]

Supplementary Table 1. The list of primers for qRT-PCR**.**

| Gene | Forward Primer | Reverse Primer |
| --- | --- | --- |
| sXBP1 | CTGAGTCCGAATCAGGTGCAG | ATCCATGGGGAGATGTTCTGG |
| CHOP | AGAACCAGGAAACGGAAACAGA | TCTCCTTCATGCGCTGCTTT |
| ATF4 | CCCTTCACCTTCTTACAACCTC | TGCCCAGCTCTAAACTAAAGGA |
| SMURF1 | AGATCCGTCTGACAGTGTTATGT | CCCATCCACGACAATCTTTGC |
| HO1 | AAGACTGCGTTCCTGCTCAAC | AAAGCCCTACAGCAACTGTCG |
| NQO1 | GAAGAGCACTGATCGTACTGGC | GGATACTGAAAGTTCGCAGGG |
| KEAP1 | CTGGAGGATCATACCAAGCAGG | GGATACCCTCAATGGACACCAC |
| NRF2 | TCAGCGACGGAAAGAGTATGA | CCACTGGTTTCTGACTGGATGT |
| Actin | CATGTACGTTGCTATCCAGGC | CTCCTTAATGTCACGCACGAT |
