# Supplementary material for: SMURF1 attenuates endoplasmic reticulum stress by promoting the degradation of KEAP1 to activate NRF2 antioxidant pathway
Source: Cell Death Dis. 2023 Jun 14;14(6):361. doi: 10.1038/s41419-023-05873-2 (PMC10267134; doi:10.1038/s41419-023-05873-2)
Supplement: Supplementary file 2 — Supplementary [file 41419_2023_5873_MOESM2_ESM.docx]

**SUPPLEMENTARY MATERIALS**

**SMURF1 attenuates endoplasmic reticulum stress by promoting the degradation of KEAP1 to activate NRF2 antioxidant pathway**

Lei Dong^1, #^, Mengchuan Xu^1, #^, Yang Li^1^, Wanting Xu^1^, Chengwei Wu^1^, Hanfei Zheng^1^, Zhenyu Xiao^1^, Guochen Sun^2^, Lei Ding^3^, Xiaobo Li^4^, Wenming Li^4^, Liying Zhou^4^, and Qin Xia^1,^ *

1: Key Laboratory of Molecular Medicine and Biological Diagnosis and Treatment (Ministry of Industry and Information Technology), School of Life Science, Beijing Institute of Technology, Beijing, China.

2: Department of Neurosurgery, The First Medical Centre, Chinese PLA General Hospital, Beijing, China. 28 Fuxing Rd, Beijing, 100853, China.

3: Key Laboratory of Carcinogenesis and Translational Research (Ministry of Education/Beijing),

Department of Anesthesiology, Peking University Cancer Hospital & Institute, Beijing 100142, China

4: BeiJing Tide Pharmaceutical Co. LTD.

**^#^**: These authors contributed equally to this work.

*****: Correspondence: qin.xia@bit.edu.cn.

**Supplementary Fig. 1 Knockdown of SMURF1 enhances UPR signaling mediated-cell death. A** The LN229 cells were treated with or without Thapsigargin (TG, 300 nM) or Tunicamycin (TM, 3 μg/mL) for 12 h, and the whole cell extracts were analyzed by western blotting with antibodies against SMURF1, GRP78 and Actin. **B** Quantification of relative intensity of SMURF1 in (**A**). **C** The LN229 and U343 cells were treated with or without TG (1 μM) or TM (10 μg/mL) for 12 h, and the relative mRNA level of SMURF1 was detected by qRT-PCR. **D** The LN229 cells were transfected with GFP plasmid for 24 h and treated with or without TG (1μM, 12 h). The whole cell extracts were analyzed by western blotting with antibodies against GFP, GRP78 and Actin. Data are presented as mean ± SD, (**p* < 0.05, ***p* < 0.01 and ****p* < 0.001).

**Supplementary Fig. 2 Knockdown of SMURF1 enhances UPR signaling mediated-cell death. A** The LN229 cells transfected with SMURF1 or scramble siRNA oligos were treated with or without TM (10 μg/mL) for 12 h, the relative mRNA levels of sXBP1, ATF4, CHOP and SMURF1 were conducted by qRT-PCR analysis. **B** The LN229 cells transfected with Flag vector or Flag-SMURF1 were treated with or without TM (10 μg/mL) for 12 h, the relative mRNA levels of sXBP1, ATF4, CHOP and SMURF1 were conducted by qRT-PCR analysis. Data are presented as mean ± SD, (**p* < 0.05, ***p* < 0.01 and ****p* < 0.001).

**Supplementary Fig. 3 SMURF1 knockdown enhances ROS production and impairs ERAD activity.** **A** The LN229 cells were transfected with GFP plasmid for 12 h, and then transfected with SMURF1 or scramble siRNA oligos for 72 h. The whole cell extracts were analyzed by western blotting with antibodies against GFP, SMURF1 and Actin. **B** The LN229 cells were transfected with GFP plasmid for 12 h, and then transfected with SMURF1 or scramble siRNA oligos for 60 h. The LN229 cells were per-treated with TG (1 μM, 6 h) and then treated with cycloheximide (CHX, 100 μg/mL) for the indicated time. The whole cell extracts were analyzed by western blotting with antibodies against GFP, SMURF1 and Actin. **C** Quantification of GFP band intensities relative to Actin. **D** The LN229 cells were transfected with GFP plasmid for 12 h, and then transfected with HA-SMURF1 or HA vector for 24 h. The LN229 cells were per-treated with TG (1 μM, 6 h) and then treated with cycloheximide (CHX, 100 μg/mL) for the indicated time. The whole cell extracts were analyzed by western blotting with antibodies against GFP, HA and Actin. **E** Quantification of GFP band intensities relative to Actin. **F** The LN229 cells were transfected with GFP plasmid for 12 h, and then transfected with SMURF1 or scramble siRNA oligos for 60 h, and treated with DMSO, NAC (2 mM) or tBHQ (20 μM) for 8 h. The whole cell extracts were analyzed by western blotting with antibodies against GFP, SMURF1 and Actin. **G** The LN229 cells were transfected with CD3-δ-YFP plasmid for 12 h, and then transfected with SMURF1 or scramble siRNA oligos for 60 h, and treated with DMSO or 4-PBA (10 μM ) for 24 h. The whole cell extracts were analyzed by western blotting with antibodies against GFP, SMURF1 and Actin. **H** Quantification of CD3-δ-YFP band intensities relative to Actin. **I** The LN229 cells were transfected with SMURF1 or scramble siRNA oligos for 72 h and treated with DMSO or NAC (2 mM) for 8 h. The whole cell extracts were analyzed by western blotting with antibodies against phospho-IRE1/IRE1, XBP1, SMURF1 and Actin. **J** Quantification of relative intensity of phospho-IRE1/IRE1 and sXBP1 in (**I**). Data are presented as mean ± SD, (**p* < 0.05, ***p* < 0.01 and ****p* < 0.001).

**Supplementary Fig. 4 SMURF1 activates NRF2 signaling pathway by promoting its nuclear import. A** The LN229 cells were transfected with SMURF1 or scramble siRNA oligos for 72 h and the relative mRNA level of NRF2 was conducted by qRT-PCR analysis. **B** The LN229 cells were transfected with SMURF1 or scramble siRNA oligos for 72 h and treated with or without TM (10 μg/ml, 12 h). The relative mRNA levels of NQO1 and HO1 were conducted by qRT-PCR analysis. **C** The LN229 cells transfected with HA vector or HA-SMURF1 were treated with or without TM (10 μg/ml, 12 h), and the relative mRNA levels of NQO1 and HO1 were performed by qRT-PCR analysis. Data are presented as mean ± SD, (**p* < 0.05, ***p* < 0.01 and ****p* < 0.001).

**Supplementary Fig. 5 SMURF1 mediates the ubiquitination and degradation of KEAP1. A** The LN229 cells were transfected with SMURF1 or scramble siRNA oligos for 72 h and the whole cell extracts were analyzed by western blotting with antibodies against KEAP1, NRF2, SMURF1 and Actin. **B** Quantification of relative intensity of KEAP1 and NRF2 in (**A**). **C** The LN229 cells were transfected with HA/Flag-SMURF1 or HA/Flag vector plasmid for 24 h and the whole cell extracts were analyzed by western blotting with antibodies against KEAP1, NRF2, HA/Flag and Actin. **D** Quantification of relative intensity of KEAP1 and NRF2 in (**C**). **E** The relative mRNA expression of KEAP1 in LN229 cells transfected with SMURF1 or scramble siRNA oligos was performed by qRT-PCR analysis. **F** The co-IP analysis of the interaction between SMURF1 and endogenous KEAP1 in the LN229 cells. **G** The LN229 cells transfected with Flag vector, Flag-SMURF1 or Flag-SMURF1-C699A were treated with TG (1 μM, 12 h), and the cytoplasmic and nuclear fractionation of NRF2, Flag, α-Tubulin and H2B were analyzed by western blotting. **H** Quantification of relative intensity of NRF2 in cytoplasm and nucleus in (**G**). **I** The LN229 cells transfected with Flag vector, Flag-SMURF1 or Flag-SMURF1-C699A were treated with or without TG (1 μM, 12 h), and the relative mRNA levels of NQO1 and HO1 were performed by qRT-PCR analysis. Data are presented as mean ± SD, (**p* < 0.05, ***p* < 0.01 and ****p* < 0.001).

**Supplementary Fig. 6 SMURF1 protects cell survival in a NRF2 dependent manner. A** The LN229 cells were transfected with NRF2 or scramble siRNA oligos for 48 h and transfected with Flag vector or Flag-SMURF1 plasmid for 24 h, then treated with TM (10 μg/mL, 12 h). The whole cell extracts were analyzed by western blotting with antibodies against Caspase3, NRF2, Flag and Actin. **B** Quantification of relative intensity of Cleaved Caspase3 in (**A**). **C** The LN229 cells overexpressing GFP were transfected with NRF2 or scramble siRNA oligos and treated with TG (1 μM, 12 h). The whole cell extracts were analyzed by western blotting with antibodies against GFP, NRF2 and Actin.
