# Supplementary material for: SMURF1 attenuates endoplasmic reticulum stress by promoting the degradation of KEAP1 to activate NRF2 antioxidant pathway
Source: Cell Death Dis. 2023 Jun 14;14(6):361. doi: 10.1038/s41419-023-05873-2 (PMC10267134; doi:10.1038/s41419-023-05873-2)
Supplement: Supplementary file 4 — Co-authors email responses [file 41419_2023_5873_MOESM4_ESM.pdf]

Co-authors' email responses

Yang Li[1], Wanting Xu[1], Chengwei Wu[1], Hanfei Zheng[1], Zhenyu Xiao[1], Guochen Sun[2], Lei Ding[3], Xiaobo Li[4], Wenming Li[4], Liying Zhou[4]

Yang Li

Coauthor confirm...
共 2 封

李杨 12:50
许梦川 ^

发件人 李杨 cherylliyang@126.com
收件人 许梦川 xumengchuan@163.com
日期 2023年05月12日 12:50

I agree to these changes.

Li Yang
Biomedical Engineering
School of life science
Beijing Institute of Technology
No. 5, Zhongguancun South Street,
Haidian District,
Beijing 100081, P. R. ChinaTel:86-15904438199
E-mail:cherylliyang@126.com

收起引文

许梦川 于 05月12日 10:35 写道

Manuscript Number: CDDIS-22-4675RR

Title: SMURF1 attenuates endoplasmic reticulum stress by promoting the degradation of KEAP1 to activate NRF2 antioxidant pathway

Authors: Lei Dong[1,#], Mengchuan Xu[1,#], Yang Li[1], Wanting Xu[1], Chengwei Wu[1], Hanfei Zheng[1], Zhenyu Xiao[1], Guochen Sun[2], Lei Ding[3], Xiaobo Li[4], Wenming Li[4], Liying Zhou[4], and Qin Xia[1,*]

Dear Li,

You have been added as a coauthor on the above manuscript. Please reply to the email confirming that you agree to these changes.

Sincerely,

Qin Xia

Wanting Xu

Coauthor confirmation
共 2 封

X 许梦川 10:37 已发送
Manuscript Number: CDDIS-22-4675RR T...

徐 徐宛婷 12:53
许梦川 ^

发件人 徐宛婷 xuwanting140910@163.com
收件人 许梦川 xumengchuan@163.com
日期 2023年05月12日 12:53

I agree to these changes.

收起引文

许梦川 于 05月12日 10:37 写道

Manuscript Number: CDDIS-22-4675RR

Title: SMURF1 attenuates endoplasmic reticulum stress by promoting the degradation of KEAP1 to activate NRF2 antioxidant pathway

Authors: Lei Dong[1,#], Mengchuan Xu[1,#], Yang Li[1], Wanting Xu[1], Chengwei Wu[1], Hanfei Zheng[1], Zhenyu Xiao[1], Guochen Sun[2], Lei Ding[3], Xiaobo Li[4], Wenming Li[4], Liying Zhou[4], and Qin Xia[1,*]

Dear Xu,

You have been added as a coauthor on the above manuscript. Please reply to the email confirming that you agree to these changes.

Sincerely,

Qin Xia

Chengwei Wu

Re: Coauthor confirmation

12:51
许梦川 ^

发件人 137814354@qq.com
收件人 许梦川 xumengchuan@163.com
日期 2023年05月12日 12:51

I aggre to these changes

收起引文

---Original---

From: "许梦川"<xumengchuan@163.com>
Date: Fri, May 12, 2023 10:40 AM
To: "137814354@qq.com" <137814354@qq.com>;
Subject: Coauthor confirmation

Manuscript Number: CDDIS-22-4675RR

Title: SMURF1 attenuates endoplasmic reticulum stress by promoting the degradation of KEAP1 to activate NRF2 antioxidant pathway

Authors: Lei Dong[1,#], Mengchuan Xu[1,#], Yang Li[1], Wanting Xu[1], Chengwei Wu[1], Hanfei Zheng[1], Zhenyu Xiao[1], Guochen Sun[2], Lei Ding[3], Xiaobo Li[4], Wenming Li[4], Liying Zhou[4], and Qin Xia[1,*]

Dear Wu,

You have been added as a coauthor on the above manuscript. Please reply to the email confirming that you agree to these changes.

Sincerely,

Qin Xia

Hanfei Zheng

5:09

Re:Coauthor confirmation

1275409614 17:06
xumengchuan ^

发件人 1275409614 1275409614@qq.com
收件人 xumengchuan xumengchuan@163.com
日期 2023年05月12日 17:06

I aggre to these changes

Zhenyu Xiao

Re:Coauthor confirmation

6120220052 18:35
许梦川 ^

发件人 6120220052
6120220052@bit.edu.cn
收件人 许梦川 xumengchuan@163.com
日期 2023年05月12日 18:35

OK

---------- Origin message ----------
>From: "许梦川" <xumengchuan@163.com>
>To: "xiaozy@bit.edu.cn" <xiaozy@bit.edu.cn>
>Subject: Coauthor confirmation
>Date: 1970-01-01 08:00:00

Manuscript Number: CDDIS-22-4675RR

Title: SMURF1 attenuates endoplasmic reticulum stress by promoting the degradation of KEAP1 to activate NRF2 antioxidant pathway

Authors: Lei Dong1, #, Mengchuan Xu1, #, Yang Li1, Wanting Xu1, Chengwei Wu1, Hanfei Zheng1, Zhenyu Xiao1, Guochen Sun2, Lei Ding3, Xiaobo Li4, Wenming Li4, Liying Zhou4, and Qin Xia1, *

Dear Professor Xiao,

You have been added as a coauthor on the above manuscript. Please reply to the email confirming that you agree to these changes.

Sincerely,

Qin Xia

Lei Ding

Coauthor confirm...
共 2 封

dingleimzk 12:42
许梦川 ^

发件人 dingleimzk dingleimzk@126.com
收件人 许梦川 xumengchuan@163.com
日期 2023年05月12日 12:42

I agree to these changes.

发自我的手机

-------- 原始邮件 --------
发件人： 许梦川 <xumengchuan@163.com>
日期： 2023年5月12日周五 上午10:42
收件人： dingleimzk@126.com
主 题： Coauthor confirmation

Manuscript Number: CDDIS-22-4675RR

Title: SMURF1 attenuates endoplasmic reticulum stress by promoting the degradation of KEAP1 to activate NRF2 antioxidant pathway

Authors: Lei Dong[1,#], Mengchuan Xu[1,#], Yang Li[1], Wanting Xu[1], Chengwei Wu[1], Hanfei Zheng[1], Zhenyu Xiao[1], Guochen Sun[2], Lei Ding[3], Xiaobo Li[4], Wenming Li[4], Liying Zhou[4], and Qin Xia[1,*]

Dear Professor Ding,

You have been added as a coauthor on the above manuscript. Please reply to the email confirming that you agree to these changes.

Sincerely,

Qin Xia

Guochen Sun

Coauthor confirm...
共 2 封

孙国臣 301医院 12:46
xumengchuan ^

发件人 孙国臣 301医院 sgc3130@126.com
收件人 xumengchuan
xumengchuan@163.com
日期 2023年05月12日 12:46

Dear xu,
I agree with the change.
Best wishes
Guochen sun

收起引文

许梦川 于 05月12日 10:43 写道

Manuscript Number: CDDIS-22-4675RR

Title: SMURF1 attenuates endoplasmic reticulum stress by promoting the degradation of KEAP1 to activate NRF2 antioxidant pathway

Authors: Lei Dong[1,#], Mengchuan Xu[1,#], Yang Li[1], Wanting Xu[1], Chengwei Wu[1], Hanfei Zheng[1], Zhenyu Xiao[1], Guochen Sun[2], Lei Ding[3], Xiaobo Li[4], Wenming Li[4], Liying Zhou[4], and Qin Xia[1,*]

Dear Professor Sun,

You have been added as a coauthor on the above manuscript. Please reply to the email confirming that you agree to these changes.

Sincerely,

Qin Xia

Xiaobo Li

Re: Coauthor confirmation

李晓博 17:40
许梦川 ^

发件人 李晓博 lixb5@tidepharm.com
收件人 许梦川 xumengchuan@163.com
日期 2023年05月12日 17:40

Dear all,

I agree to these changes.

Xiaobo

收起引文

-----Original Messages-----
From:"许梦川" <xumengchuan@163.com>
Sent Time:2023-05-12 10:45:29 (Friday)
To: "lixb5@tidepharm.com" <lixb5@tidepharm.com>
Cc:
Subject: Coauthor confirmation

Manuscript Number: CDDIS-22-4675RR

Title: SMURF1 attenuates endoplasmic reticulum stress by promoting the degradation of KEAP1 to activate NRF2 antioxidant pathway

Authors: Lei Dong[1,#], Mengchuan Xu[1,#], Yang Li[1], Wanting Xu[1], Chengwei Wu[1], Hanfei Zheng[1], Zhenyu Xiao[1], Guochen Sun[2], Lei Ding[3], Xiaobo Li[4], Wenming Li[4], Liying Zhou[4], and Qin Xia[1,*]

Dear Professor Li,

You have been added as a coauthor on the above manuscript. Please reply to the email confirming that you agree to these changes.

Sincerely,

Qin Xia

Wenming Li

Coauthor confirmation

共 2 封

L 许梦川 10:46 已发送

Manuscript Number: CDDIS-22-4675RR T...

李 李文明 12:51

许梦川 ^

发件人 李文明 liwm@tidepharm.com

收件人 许梦川 xumengchuan@163.com

日期 2023年05月12日 12:51

Dear Dr. Xu

I confirm that I agree to be added as a co-author on the manuscript which number is CDDIS-22-4675RR.

Thanks for your kindness!

Wenming Li

在 2023年5月12日 10:46，许梦川 <xumengchuan@163.com> 写道:

> Manuscript Number: CDDIS-22-4675RR
>
> Title: SMURF1 attenuates endoplasmic reticulum stress by promoting the degradation of KEAP1 to activate NRF2 antioxidant pathway
>
> Authors: Lei Dong[1, #], Mengchuan Xu[1, #], Yang Li[1], Wanting Xu[1], Chengwei Wu[1], Hanfei Zheng[1], Zhenyu Xiao[1], Guochen Sun[2], Lei Ding[3], Xiaobo Li[4], Wenming Li[4], Liying Zhou[4], and Qin Xia[1, *]
>
> Dear Professor Li,
>
> You have been added as a coauthor on the above manuscript. Please reply to the email confirming that you agree to these changes.
>
> Sincerely,
>
> Qin Xia

Liying Zhou

Z Zhouly [illegible]

'许梦川' ^

发件人 Zhouly zhouly@tidepharm.com

收件人 '许梦川' xumengchuan@163.com

日期 2023年05月12日 14:56

Dear Qin Xia,

Thanks for your email. I agree these changes.

Best Regards,

Liying Zhou

发件人: 许梦川 <xumengchuan@163.com>
发送时间: 2023年5月12日 10:47
收件人: zhouly@tidepharm.com
主题: Coauthor confirmation

Manuscript Number: CDDIS-22-4675RR

Title: SMURF1 attenuates endoplasmic reticulum stress by promoting the degradation of KEAP1 to activate NRF2 antioxidant pathway

Authors: Lei Dong[1, #], Mengchuan Xu[1, #], Yang Li[1], Wanting Xu[1], Chengwei Wu[1], Hanfei Zheng[1], Zhenyu Xiao[1], Guochen Sun[2], Lei Ding[3], Xiaobo Li[4], Wenming Li[4], Liying Zhou[4], and Qin Xia[1, *]

Dear Professor Zhou,

You have been added as a coauthor on the above manuscript. Please reply to the email confirming that you agree to these changes.

Sincerely,

Qin Xia
