# Supplementary figures and images for: SMURF1 attenuates endoplasmic reticulum stress by promoting the degradation of KEAP1 to activate NRF2 antioxidant pathway
Source: Cell Death Dis. 2023 Jun 14;14(6):361. doi: 10.1038/s41419-023-05873-2 (PMC10267134; doi:10.1038/s41419-023-05873-2)

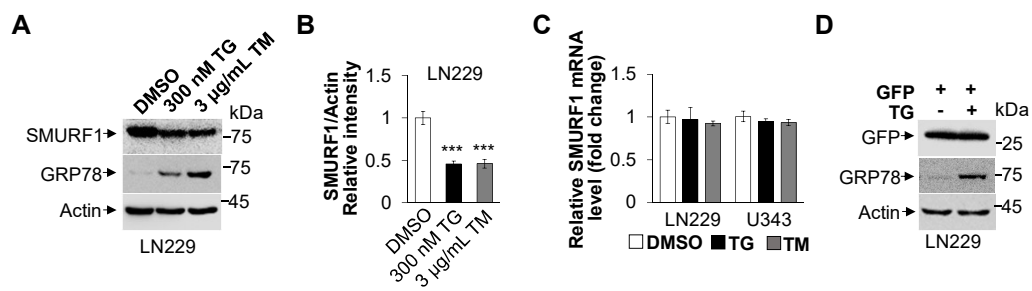

Supplement: Supplementary file 5 — Supplementary Figure 1 [file 41419_2023_5873_MOESM5_ESM.pdf]

**A**

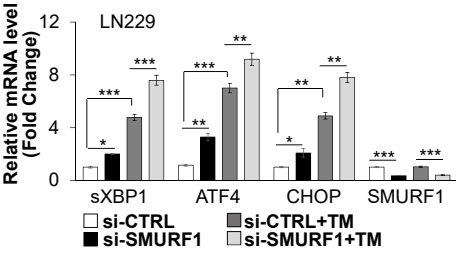

**B**

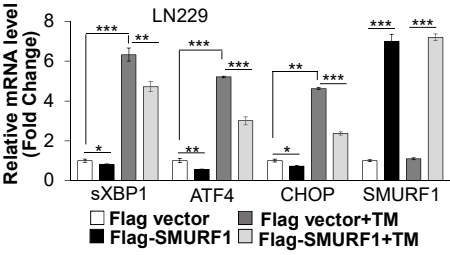

Supplement: Supplementary file 6 — Supplementary Figure 2 [file 41419_2023_5873_MOESM6_ESM.pdf]

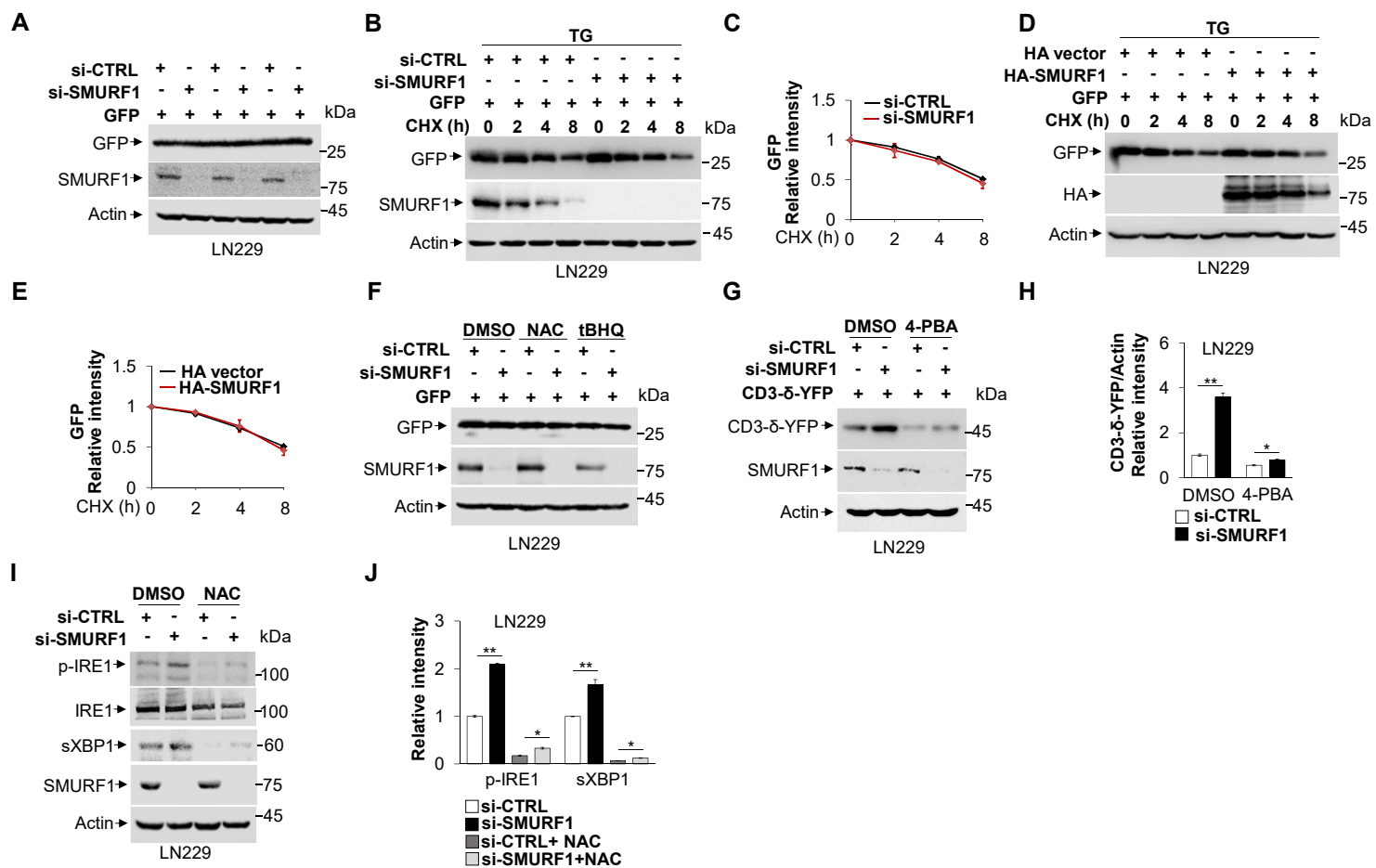

Supplement: Supplementary file 7 — Supplementary Figure 3 [file 41419_2023_5873_MOESM7_ESM.pdf]

**A**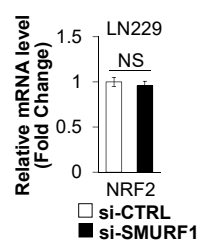**B**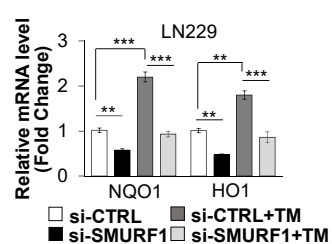**C**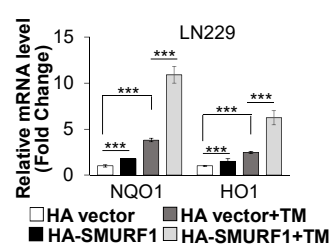

Supplement: Supplementary file 8 — Supplementary Figure 4 [file 41419_2023_5873_MOESM8_ESM.pdf]

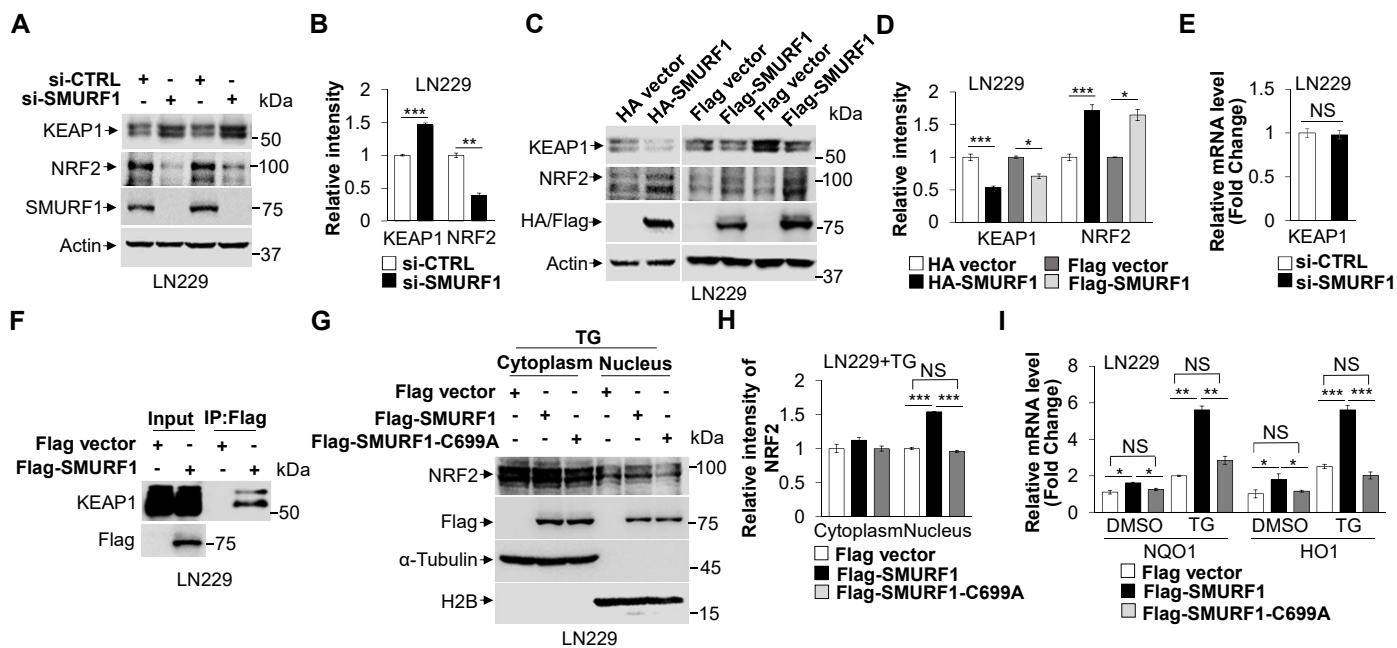

Supplement: Supplementary file 9 — Supplementary Figure 5 [file 41419_2023_5873_MOESM9_ESM.pdf]

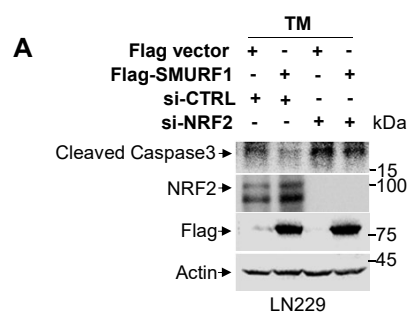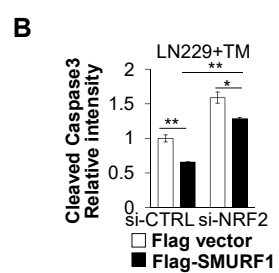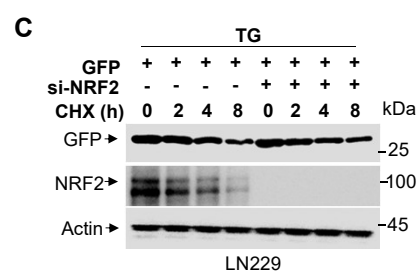

Supplement: Supplementary file 10 — Supplementary Figure 6 [file 41419_2023_5873_MOESM10_ESM.pdf]
