# Supplementary material for: SMURF1 attenuates endoplasmic reticulum stress by promoting the degradation of KEAP1 to activate NRF2 antioxidant pathway
Source: Cell Death Dis. 2023 Jun 14;14(6):361. doi: 10.1038/s41419-023-05873-2 (PMC10267134; doi:10.1038/s41419-023-05873-2)

Fig. 1A

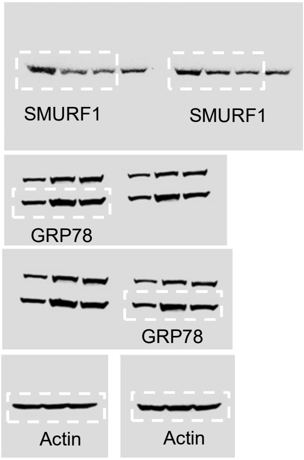

Fig. 1E

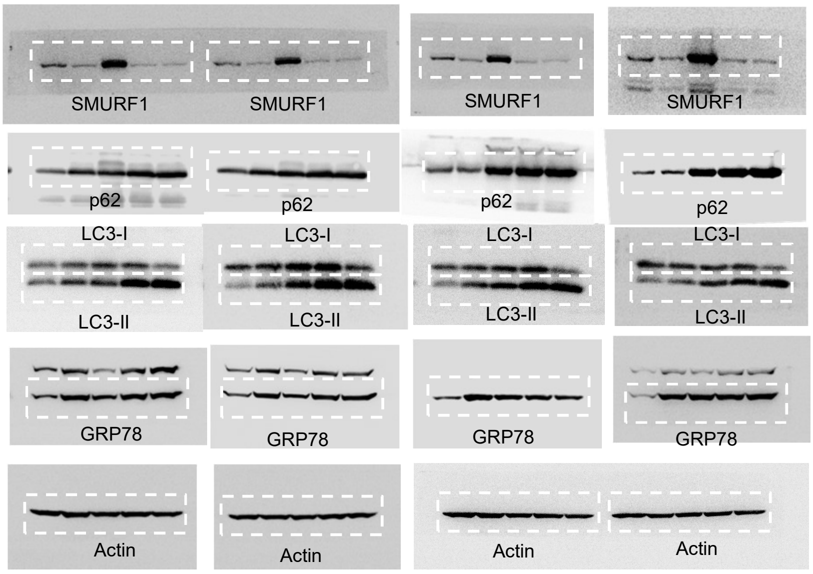

Fig. 1C

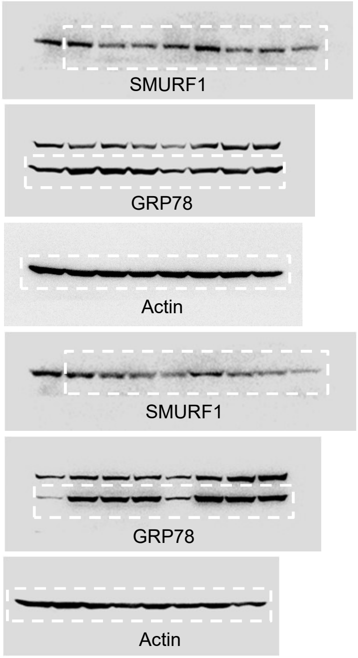

Fig. 2A

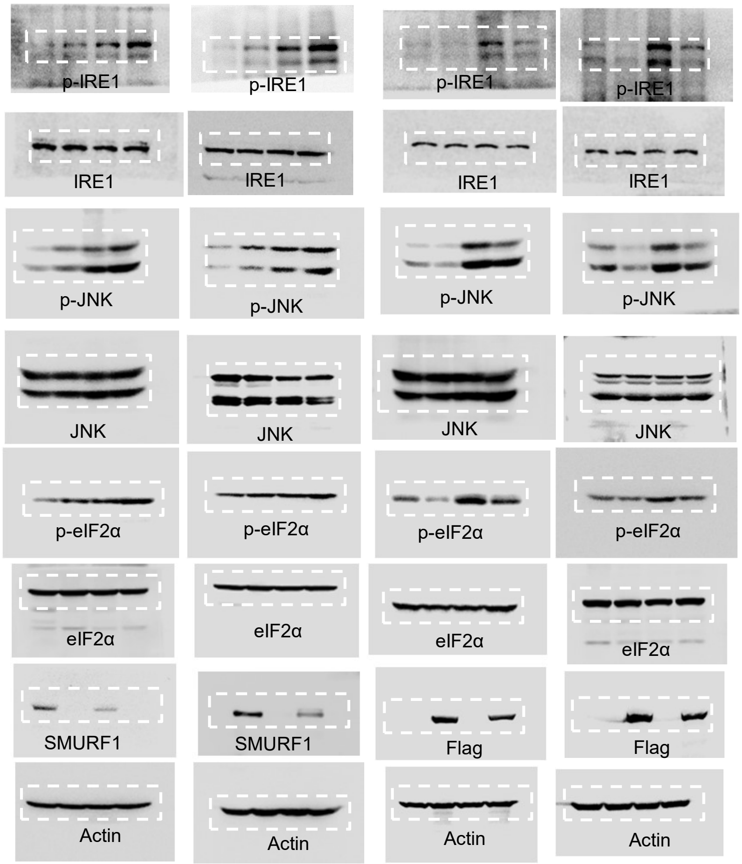

Fig. 1G

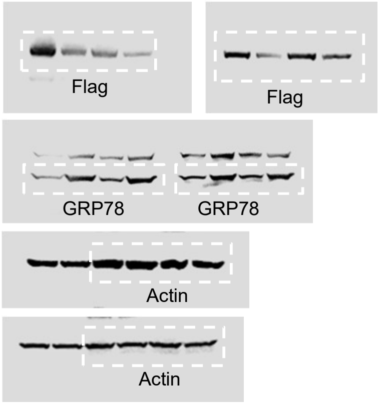

Fig. 2D

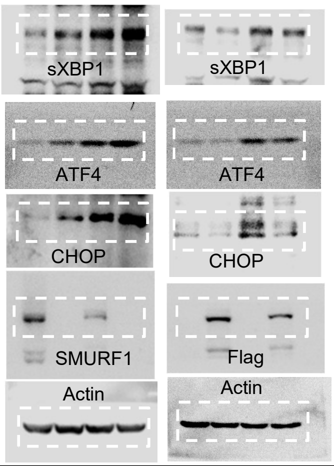

Fig. 2I

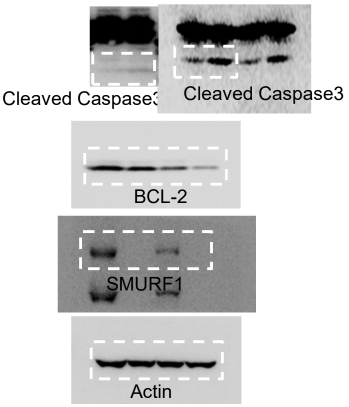

Fig. 2K

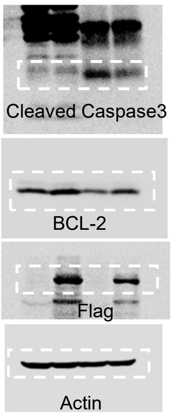

Fig. 2O

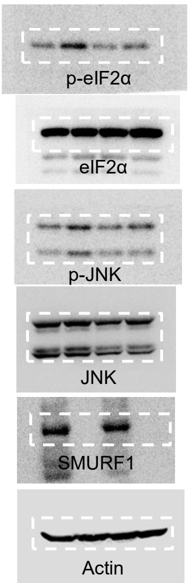

Fig. 2Q

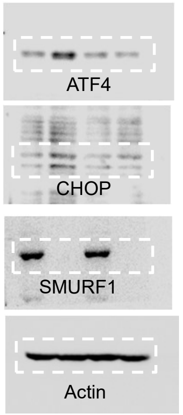

Fig. 3E

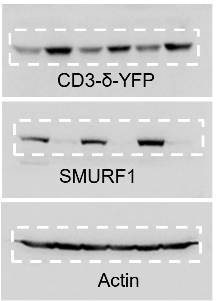

Fig. 3H

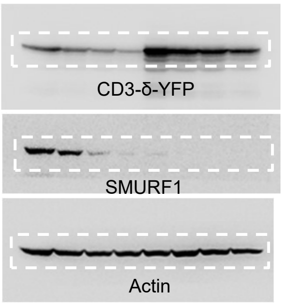

Fig. 3J

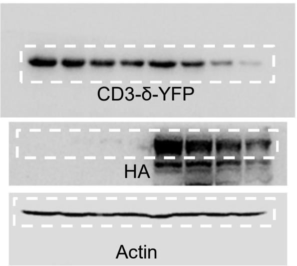

Fig. 3L

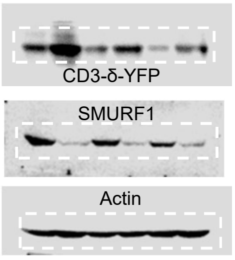

Fig. 4A

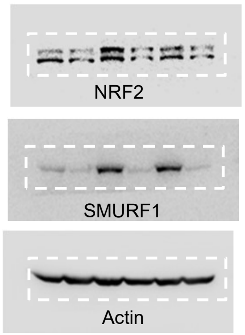

Fig. 4C

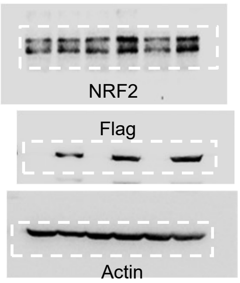

Fig. 4E

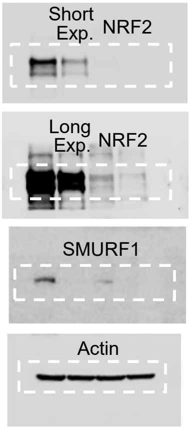

Fig. 4G

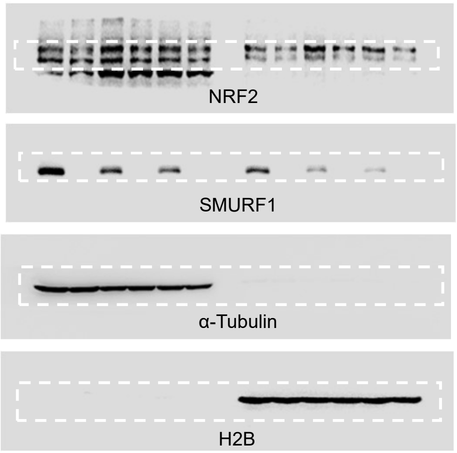

Fig. 4J

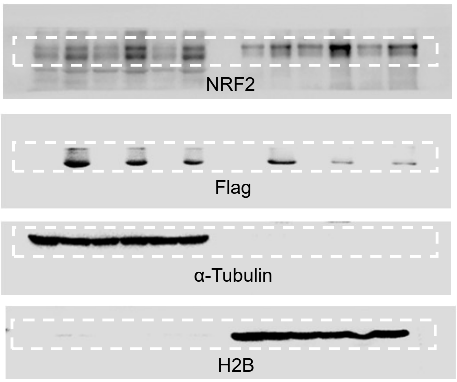

Fig. 5A

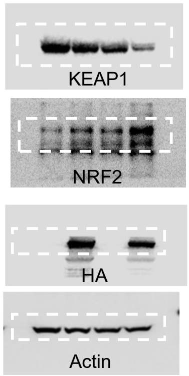

Fig. 5C

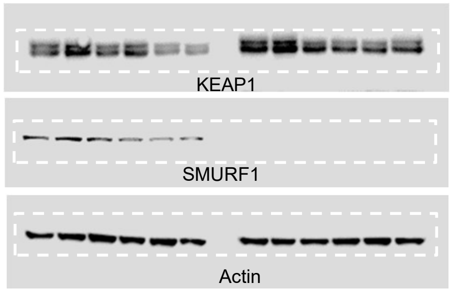

Fig. 5E

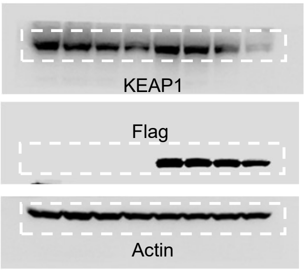

Fig. 5G

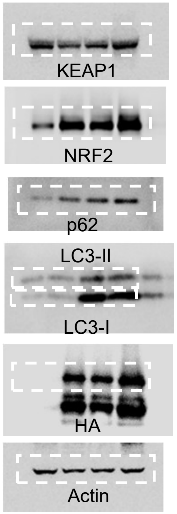

Fig. 5I

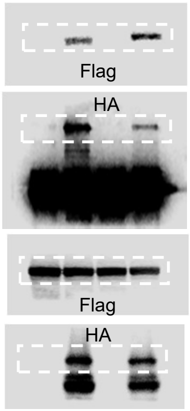

Fig. 5K

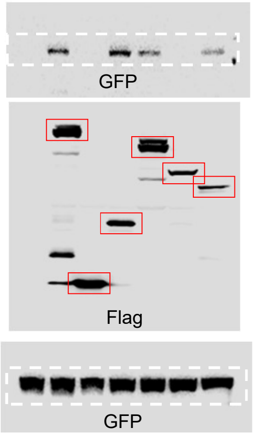

Fig. 5L

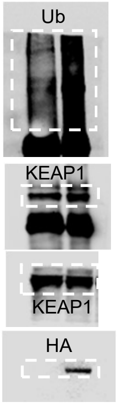

Fig. 5M

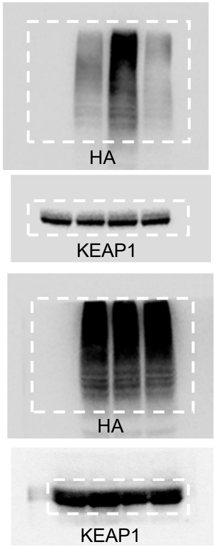

Fig. 6F

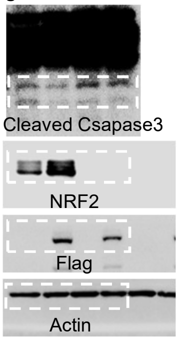

Fig. 6O

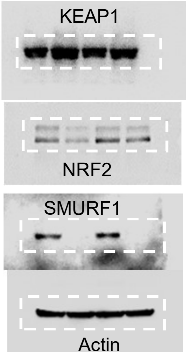

Fig. 6A

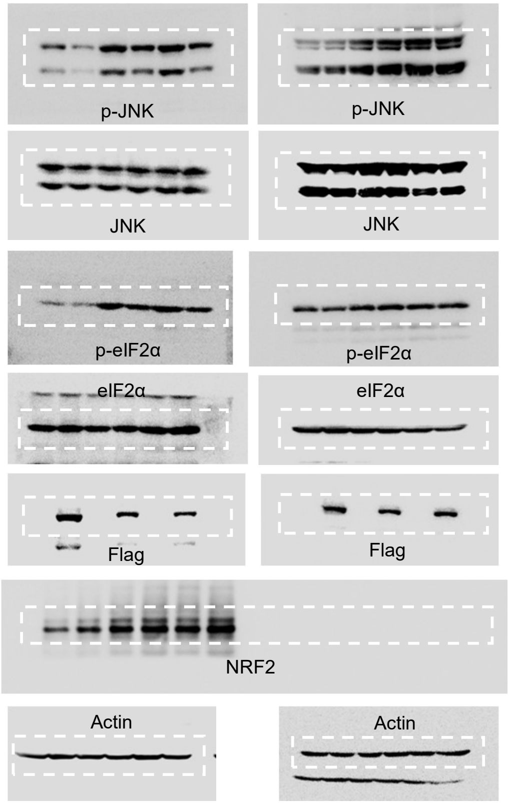

Fig. 6I

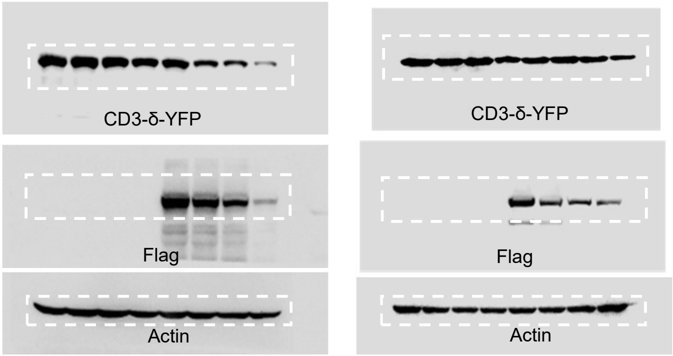

Fig. S1A

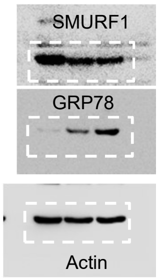

Fig. S1D

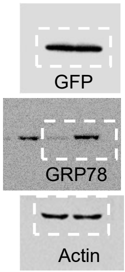

Fig. S3A

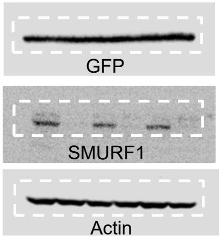

Fig. S3B

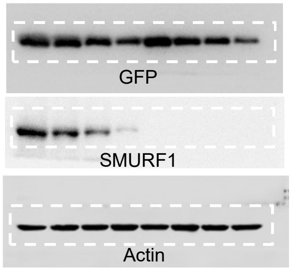

Fig. S3D

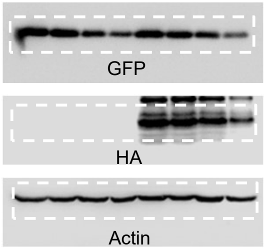

Fig. S3F

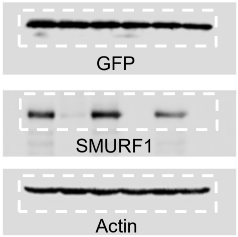

Fig. S3G

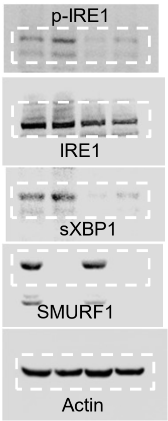

Fig. S5A

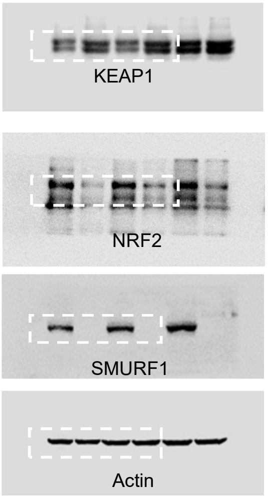

Fig. S5C

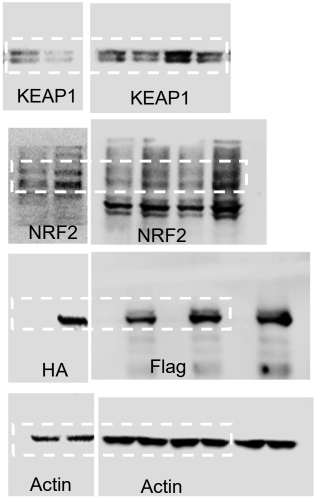

Fig. S5F

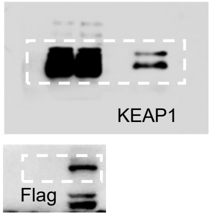

Fig. S5G

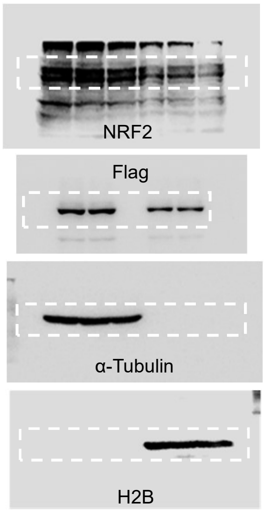

Fig. S6A

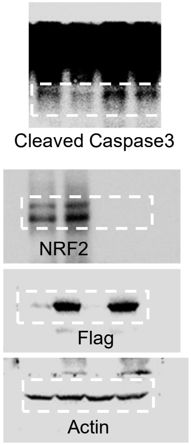

Fig. S6C

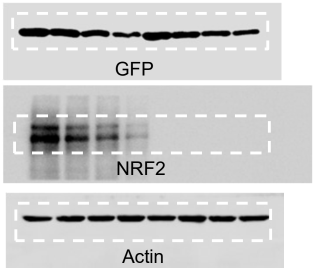

Supplement: Supplementary file 11 — Original Data File [file 41419_2023_5873_MOESM11_ESM.pdf]
